# Molecular chaperone RAP interacts with LRP1 in a dynamic bivalent mode and enhances folding of ligand-binding regions of other LDLR family receptors

**DOI:** 10.1016/j.jbc.2021.100842

**Published:** 2021-05-29

**Authors:** Ekaterina Marakasova, Philip Olivares, Elena Karnaukhova, Haarin Chun, Nancy E. Hernandez, James H. Kurasawa, Gabriela U. Hassink, Svetlana A. Shestopal, Dudley K. Strickland, Andrey G. Sarafanov

**Affiliations:** 1 Center for Biologics Evaluation and Research, US Food and Drug Administration, Silver Spring, Maryland, USA; 2 Center for Vascular and Inflammatory Diseases, University of Maryland School of Medicine, Baltimore, Maryland, USA

**Keywords:** low-density lipoprotein (LDL), factor VIII (FVIII), molecular chaperone, protein expression, protein folding, LDL-receptor related protein-associated protein (RAP), ApoE3, apolipoprotein E3 protein, ASPP, ammonium sulfate/potassium phosphate (buffer), CD, circular dichroism, CR, complement-type repeat, ER, endoplasmic reticulum, EDTA, ethylenediaminetetraacetic acid, K_D_, dissociation constant, LDL, low-density lipoprotein, LDLR, low-density lipoprotein receptor, LRP1, low-density receptor-related protein 1, RAP, LDL-receptor related protein-associated protein, RU, response units, SE-FPLC, size-exclusion fast protein liquid chromatography, SPR, surface plasmon resonance, vLDLR, very low-density lipoprotein receptor

## Abstract

The low-density lipoprotein receptor (LDLR) family of receptors are cell-surface receptors that internalize numerous ligands and play crucial role in various processes, such as lipoprotein metabolism, hemostasis, fetal development, etc. Previously, receptor-associated protein (RAP) was described as a molecular chaperone for LDLR-related protein 1 (LRP1), a prominent member of the LDLR family. We aimed to verify this role of RAP for LRP1 and two other LDLR family receptors, LDLR and vLDLR, and to investigate the mechanisms of respective interactions using a cell culture model system, purified system, and *in silico* modelling. Upon coexpression of RAP with clusters of the ligand-binding complement repeats (CRs) of the receptors in secreted form in insect cells culture, the isolated proteins had increased yield, enhanced folding, and improved binding properties compared with proteins expressed without RAP, as determined by circular dichroism and surface plasmon resonance. Within LRP1 CR-clusters II and IV, we identified multiple sites comprised of adjacent CR doublets, which provide alternative bivalent binding combinations with specific pairs of lysines on RAP. Mutational analysis of these lysines within each of isolated RAP D1/D2 and D3 domains having high affinity to LRP1 and of conserved tryptophans on selected CR-doublets of LRP1, as well as *in silico* docking of a model LRP1 CR-triplet with RAP, indicated a universal role for these residues in interaction of RAP and LRP1. Consequently, we propose a new model of RAP interaction with LDLR family receptors based on switching of the bivalent contacts between molecules over time in a dynamic mode.

The receptors from the low-density lipoprotein receptor (LDLR) family are expressed in many tissues where they recognize various dissimilar ligands involved in numerous biological processes. In humans, these receptors are represented by LDLR, LDLR-related protein 1 (LRP1), very low-density lipoprotein receptor (vLDLR), ApoER2, LRP2, LRP1B, and LRP4 (1). In circulation, LDLR, LRP1, and vLDLR are responsible for endocytosis of various proteins and lipoproteins (2), and misfunction of these receptors may result in atherosclerotic disease and other abnormalities. In other tissues, LRP1, vLDLR, and ApoER2 are involved in cell signaling and tissue remodeling, and all are implicated in Alzheimer's disease (3, 4). Other pathological processes implicating the LDLR family receptors involve cardiovascular diseases, type 2 diabetes, obesity, Parkinson's disease, and others (5, 6, 7). Better knowledge of respective receptor–ligand interactions is important for understanding these processes and fundamental discoveries in the future.

The LDLR family receptors are composed of the same domain types serving specific functional roles. The ligand-binding function is generally served by highly homologous complement-type repeats (CRs) organized in clusters (7, 8). Relatively simple in structure, LDLR, vLDLR, ApoER2, and LRP4 have one cluster formed by seven to eight CRs, whereas other receptors have four clusters of CRs formed by similar or larger numbers of repeats. In a prominent member of the family, LRP1, there are two major ligand-binding clusters termed II and IV, less significant clusters for ligand binding include cluster III and cluster I (Fig. S1), which is known to participate with cluster II in binding of only one ligand, activated forms of alpha-2-macroglobulin (9, 10). LRP1 also binds triglyceride-rich particles, fibronectin, matrix proteases, and blood clotting factors with a total number of more than 40 of disparate ligands (9).

Each CR domain is formed from ∼40 amino acids and connected to an adjacent CR domain with a flexible linker that, in the case of LRP1, is composed of three to ten amino acids. Each CR domain's structure is enforced by three internal disulfide bonds formed from six conserved cysteines and by coordination of a Ca^2+^ ion with four conserved acidic residues (11, 12). During ligand binding, the conserved acidic residues and an aromatic residue interact with ε-amino group and aliphatic portion of a “critical” lysine of the ligand, respectively, and additional interface residues provide weaker binding energy. This mechanism was described for the interactions of vLDLR with a human rhinovirus (13), ApoER2 with reelin (14), LDLR with receptor-associated protein (RAP), and proposed to be common for the ligands' recognition by all LDLR family receptors (15).

RAP was described as a molecular chaperone for LRP1 and antagonist for its interactions with ligands (16, 17, 18). This indicates that RAP may also serve the chaperone function for other LDLR family receptors, which is supported by several studies (19, 20, 21, 22). In interaction with LRP1, RAP was proposed to bind the CR domains of the newly synthesized receptor to assist their folding, prevent premature binding to other ligands, and deliver the molecule to the cell surface (23). Several studies demonstrated that interaction of RAP and LRP1 (and also LDLR) involves formation of a complex between two “critical” lysines of RAP and a doublet of adjacent CR domains (15, 24, 25, 26), where each lysine docks to a single CR domain; and these binding events provide an additive (avidity) effect enforcing the interaction (15, 23, 26). Such a mode of interaction was termed “bivalent” regarding the interaction of LRP1 and an isolated fragment of RAP composed of its D1 and D2 domains (D1/D2) bearing a high-affinity site for binding to LRP1. On D1/D2, the “critical” lysines K60 and K191 are located on D1 and D2, respectively; both these domains are connected with a flexible linker, and therefore both lysines have mutual flexibility (23). In contrast, the two “critical” lysines of the second high-affinity LRP1-binding site of RAP, K256, and K270 are located on the single domain (D3), which has a rigid structure with minimal mutual flexibility of the lysines. The role of these lysines was established by testing interactions of isolated D3 with LRP1 and its CR-doublet 5–6 (26) and also CR-doublet 3–4 of LDLR (15); therefore the interaction of D3 with both full-size LRP1 and LDLR also corresponds to the bivalent mode. Thus, each of the two sites of RAP can bivalently interact with LRP1; however, it is unclear how these binding events are mutually coordinated during the interaction of both molecules.

In LRP1, the binding sites for RAP are located within clusters II, III, and IV. Each of the isolated clusters is able to interact with RAP with affinity comparable to that for the full-length receptor (K_D_ 1–5 nM) (23, 27, 28, 29). Within the clusters II and III, the majority of CR doublets were shown to bind RAP and its isolated D1/D2 and D3 fragments with comparable affinities (24, 27, 28, 30). Within cluster IV, the active binding CR doublets have not yet been identified; however, previous studies indicated that the majority of its CRs are capable of binding LRP1 (30, 31). Notably, the data show that the absence of the conserved aromatic residue in any domain of a CR doublet (Fig. S1) correlates with its inability to bind RAP as shown for CRs 1–2 (cluster I), 9–10 (cluster II), and 19–20 (cluster III). Thus, these studies show that numerous LRP1 sites are capable to facilitate bivalent binding combinations with RAP. However, like the sites on RAP, it is unclear how these sites in LRP1 are coordinated during its interaction with RAP.

Until now, the chaperone function of RAP has been supported only for LRP1, LRP2, and vLDLR, but not LDLR. Indeed, (i) disruption of the RAP gene in mouse model resulted in impairing the expression of these receptors, except LDLR (21, 22); (ii) cotransfection of the RAP gene in cell culture facilitated expression of recombinant vLDLR and LRP1, but not LDLR (20), and (iii) coexpression of RAP and the LRP1 exodomain in cell culture resulted in increase of the latter's yield (19). At the same time, expression of recombinant CR fragments of LRP1 and LDLR in cell culture yielded relatively low amounts of correctly folded proteins (28, 32, 33) indicating requirement of a folding factor. Notably, the affinity of RAP for LDLR was found to be similar (32) or weaker (34) than that for LRP1.

Due to the ability of RAP to interact with all LDLR family receptors, it is used as a model ligand to study their interactions with other ligands based on similarity of the respective mechanisms. In particular, the bivalent binding mode has also been described for the interactions of LRP1 with blood coagulation factor VIII (FVIII) (35) and plasminogen-activator inhibitor 1 (PAI-1) (36). In our study, we aimed to characterize interactions of RAP with selected LDLR family receptors in several model systems on to obtain a deeper insight into these mechanisms. Our basic approach was to test RAP interactions with ligand-binding fragments of LRP1, LDLR, and vLDLR within living cells upon the coexpression of RAP with proteins (Fig. 1*A*). For this, we used an insect-cell-based platform, which is not capable of providing a relevant folding factor for the receptors as resulted in production of their fragments mostly in misfolded forms (28, 32, 33). To dissect the mechanism of RAP and LRP1 interaction to smaller molecular determinants (Fig. 1*B*), we tested binding of their fragments, including mutated variants, using a purified system and *in silico* modeling. The resulting data support the function of RAP as a folding chaperone for the tested receptors and a bivalent mechanism of the interactions, which occur in dynamic mode.

**Figure 1:**
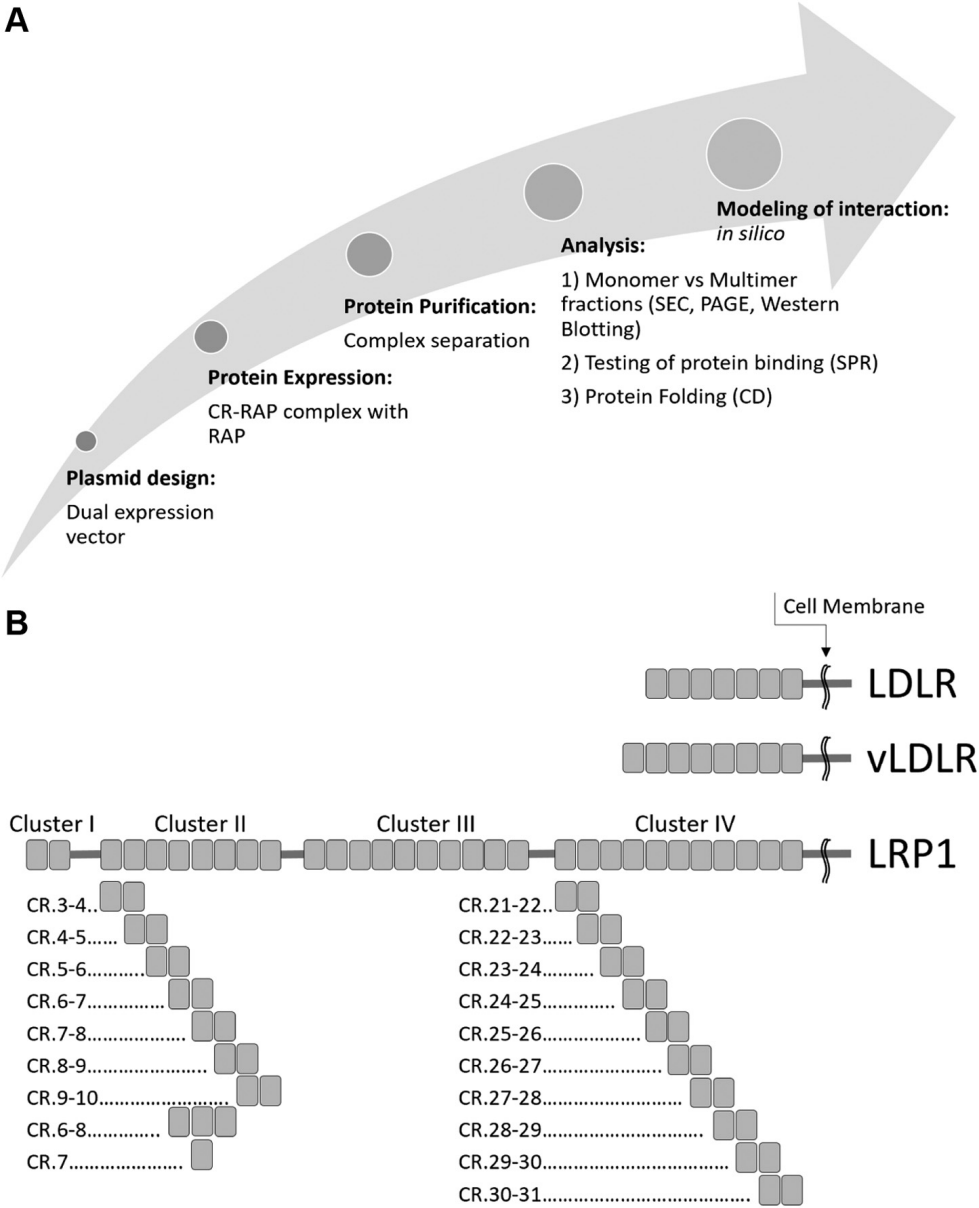
**Experimental strategy for testing interactions of RAP with LRP1, LDLR, and vLDLR.***A*, testing interactions of the receptors' CR-fragments with RAP. *B*, organization of CRs (*gray rectangles*) in the receptors and expression of the respective CR fragments: LDLR (cluster of seven CRs), vLDLR (cluster of eight CRs), LRP1 clusters II (CR.3–10) and IV (CR.21–31), and CR fragments of these clusters. CD, circular dichroism; SEC, size-exclusion chromatography; SPR, surface plasmon resonance

## Results

### Coexpression with RAP results in increased LRP1 cluster II yield

**Figure 2:**
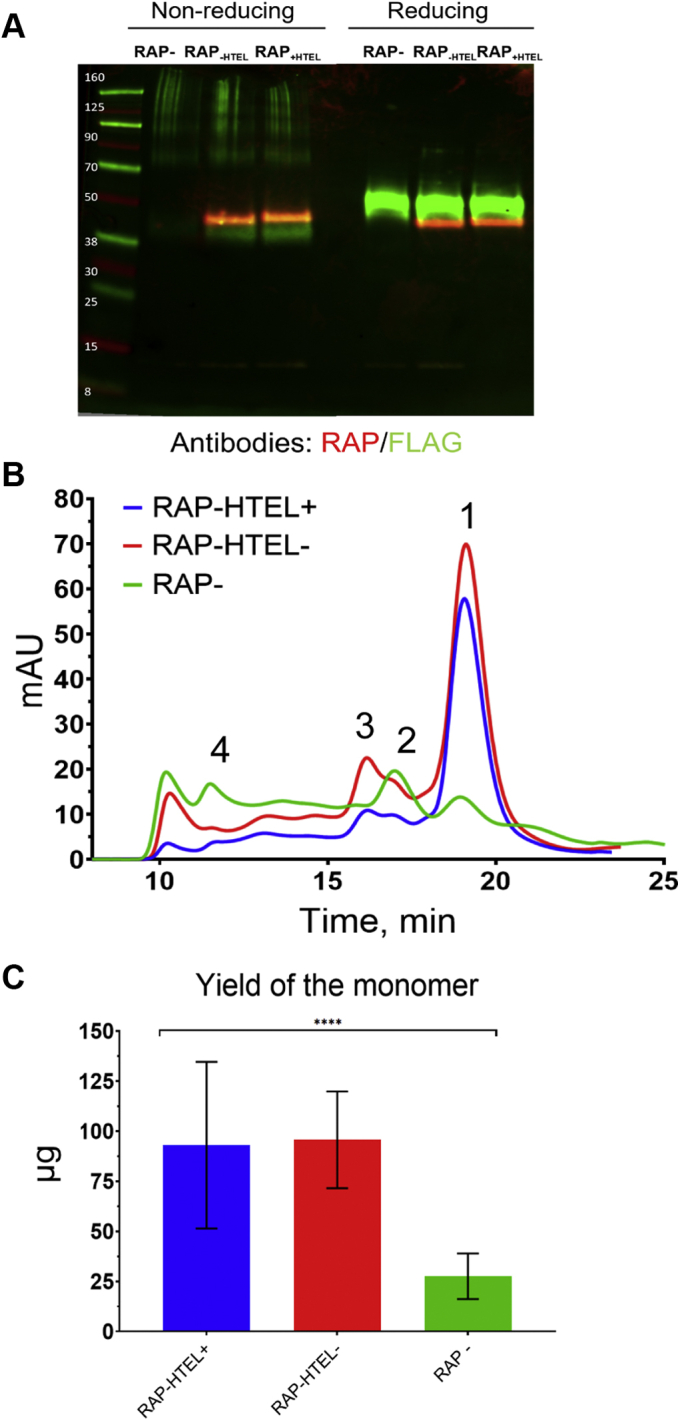
**Coexpression with RAP results in increased yield of LRP1 cluster II monomer yield**. *A*, western blotting analysis of the expression medium: RAP−, LRP1 cluster II expressed without RAP; RAP (-HTEL), LRP1 cluster II coexpressed with RAP not having HTEL signal for ER retention; RAP (+HTEL), LRP1 cluster II coexpressed with RAP having the HTEL signal. Staining in red: detection of RAP using anti-RAP antibodies; staining in green: detection of LRP1 cluster II using anti-FLAG tag antibodies. In both RAP coexpressed samples, LRP1 cluster II was cosecreted in a tight complex with RAP, and its removal required using a high salt/imidazole EDTA-containing wash at the Ni-column (first step of protein purification). *B*, separation of LRP1 cluster II molecular forms by SE-FPLC: peak 1 corresponds to protein monomer (correctly folded protein); peaks 2, 3, and 4 correspond to the dimers, trimers, and multimers (randomly folded protein). *C*, yield of LRP1 cluster II monomer (μg ± SD) from 200 ml of cell culture medium upon purification (Experimental procedures). *p* value < 0.0001 (∗∗∗∗).

First, we tested coexpression of RAP and LRP1 cluster II, based on the previously reported increase of LRP1 exodomain production upon coexpression with RAP in mammalian cell culture (19). The goal was to verify the suitability of an insect-cell-based (baculovirus) system for testing RAP and LDLR family receptor fragments' interactions within living cells. In particular, insect cells are not capable of providing, at least in sufficient amount, a factor to facilitate the folding of the CR domains, which results in protein secretion mostly in misfolded multimeric forms due to mislinking of the conserved cysteines (28, 32, 33). We considered such a background to be favorable for testing an effect of RAP on protein expression. Following our strategy (Fig. 1), we prepared three dual-gene baculovirus-based constructs, which coded for LRP1 cluster II and human RAP (i) with or (ii) without an ER retention signal HTEL (an insect variant of the mammalian HNEL signal) to test possibility of recycling RAP within cell and (iii) not containing the RAP gene (Table S1), driving protein secretion into the media. Upon their expression, the Sf9 cells culture media was analyzed by western blotting using anti-RAP and anti-FLAG tag (fused to cluster II) antibodies. The nonreducing gel conditions data indicated that RAP coexpression facilitated expression of correctly folded LRP1 cluster II based on increased yield of its monomer (∼38 kDa) (Fig. 2*A*). The protocol for protein purification, in particular the removal of RAP cosecreted with LRP1 cluster II in a tight complex, independent on the presence or absence of the HTEL signal, was developed. Specifically, removal of RAP from the complex with LRP1 cluster II protein bound to Ni-column required using a high salt/imidazole-EDTA washing buffer, as it was not possible to achieve using a standard medium salt/imidazole buffer commonly used for purification of His-tagged proteins. Consistent with gel analysis, the protein molecular weight profiling by size-exclusion fast protein liquid chromatography (SE-FPLC) showed more than 4-fold prevalence of the cluster monomer (correctly folded protein) over its multimers for both coexpressed RAP variants, compared with protein expressed in the absence of RAP (Fig. 2*B*). Both reducing gel conditions and protein monomer yield (∼0.5 mg/L, average from two experiments) upon the coexpression with either RAP/no HTEL or RAP/HTEL showed no practical difference in the expression levels (Fig. 2*C*). Thus, RAP coexpression resulted in significantly higher yield of LRP1 cluster II monomer, independent of the presence or absence of the HTEL tag on RAP.

### Coexpression with RAP improves folding and binding properties of LRP1 cluster II

In this and further experiments, our CD assessment was aimed at the evaluation of the possible impact of RAP coexpression on the folding of CR fragments by comparison of percentage of the secondary structure elements, in particular, evaluating the content of the unordered fraction. Whereas determination of the secondary structure elements by CDPro does not provide absolute structural information, this approach is useful for a comparison of the secondary structure between closely related proteins, such as CR fragments expressed with or without RAP in this study. Both preparations of LRP1 cluster II exhibited characteristic CD spectra with a strong negative CD extremum around 200 nm being suggestive of a high content of β-structures, high content of unordered elements, and low content of α-helices (Table 1). Evaluation of the secondary structure elements in each protein by CDPro supported the expectation. As seen in Table 1, the content of α-helical elements appears to be very low, 4.3%–7.1%, whereas the contents of β-structures were significantly higher, varying from 35% to 60%. Notably, the percentage of unordered structure elements in LRP1 cluster II coexpressed with RAP was found to be lower than in protein expressed without RAP.

**Table 1:** Protein-folding-related parameters by CD

Protein^a^	MW^b^ (Da)	AA^c^ (n)	D^d^ (n)	D+E^e^ (n)	W^f^ (n)	C^g^ (n)	α-helix (%)	β-sheet (%)	β-turn (%)	Unrd^h^ (%)	∼200 nm^i^ peak (nm)	ΔCD^j^ (%)	∼230 nm^k^ peak (nm)
LDLR cluster (7 CRs) (RAP+)^l^	42,768	334	48	68	6	42	7.1	35.6	24.4	32.9	200/201	33.4	Yes
LDLR cluster (7 CRs) (RAP−)^m^	42,768	334	48	68	6	42	5.6	36.6	23.5	34.3	200/201	19.1	Yes
vLDLR cluster (8 CRs) (RAP+)	41,517	355	52	81	6	48	8.3	35.2	24.3	32.2	202/203	17.6	Yes
vLDLR cluster (8 CRs) (RAP−)	41,517	355	52	81	6	48	8.4	33.9	22.1	35.6	203/203	6.2	No
LRP1 cluster II (8 CRs) (RAP+)	38,036	349	47	66	7	48	6.2	24.0	14.9	54.9	200/201	20.0	Yes
LRP1 cluster II (8 CRs) (RAP−)	38,036	349	47	66	7	48	6.5	20.0	14.9	58.6	2001/202	9.4	No
LRP1 CR. 6–8 (RAP+)	20,903	190	28	35	3	18	6.1	32.7	25.6	35.6	199/201	33.3	Yes
LRP1 CR.6–8 (RAP−)	17,249	156	18	27	3	18	5.1	31.6	26.2	37.1	199/201	24.1	Yes
LRP1 CR. 6–7 (RAP+)	15,507	141	21	25	2	12	6.1	26.8	24.7	42.4	198/200	38.6	Yes
LRP1 CR 6–7 (RAP−)	15,507	141	21	25	2	12	6.2	20.5	26.2	47.1	198/200	23.2	Yes
LRP1 CR.7–8 (RAP+)	16,459	150	23	29	2	12	7.0	30.4	24.1	38.5	199/200	28.1	Yes
LRP1 CR.7–8 (RAP−)	16,459	150	23	29	2	12	6.4	31.1	21.6	40.9	200/200	11.7	No
LRP1 CR.30–31 (RAP+)	16,618	149	24	31	1	12	6.2	34.8	23.3	35.7	200/201	13.2	No
LRP1 CR.30–31 (RAP−)	16,618	149	24	31	1	12	5.7	33.9	24.0	36.4	200/201	7.5	No
LRP1 CR.7 (RAP+)	11,063	101	16	19	1	6	4.8	31.1	24.6	42.5	198/200	19.4	No
LRP1 CR.7 (RAP−)	11,063	101	16	19	1	6	4.3	30.4	24.1	44.2	198/200	9.7	No

a Protein information corresponds to UniProt ID: P01130 (LDLR), P98155 (vLDLR) and Q07954 (LRP1).

b Molecular weight (MW).

c Number of amino acid residues (AA).

d Number of aspartic acid residues (D).

e Number of aspartic acid and glutamic acid residues (D + E).

f Number of tryptophan residues (W).

g Number of cysteine residues (C).

h Unordered structure elements (Unrd).

i Main negative CD extremum at ∼200 nm: position before and (/) after EDTA addition to the sample in Ammonium Sulfate/Potassium Phosphate (ASPP) buffer to concentration of 5 mM.

j Change in intensity of the negative peak at ∼200 nm upon the addition of EDTA (ΔCD) as illustrated in Figure S2*B*.

k Presence of ∼230 nm peak related to number and conformation of tryptophanes (W) and disulfide bonds (C).

l Protein co-expressed with RAP (RAP+).

m Protein expressed without RAP (RAP−).

**Figure 3:**
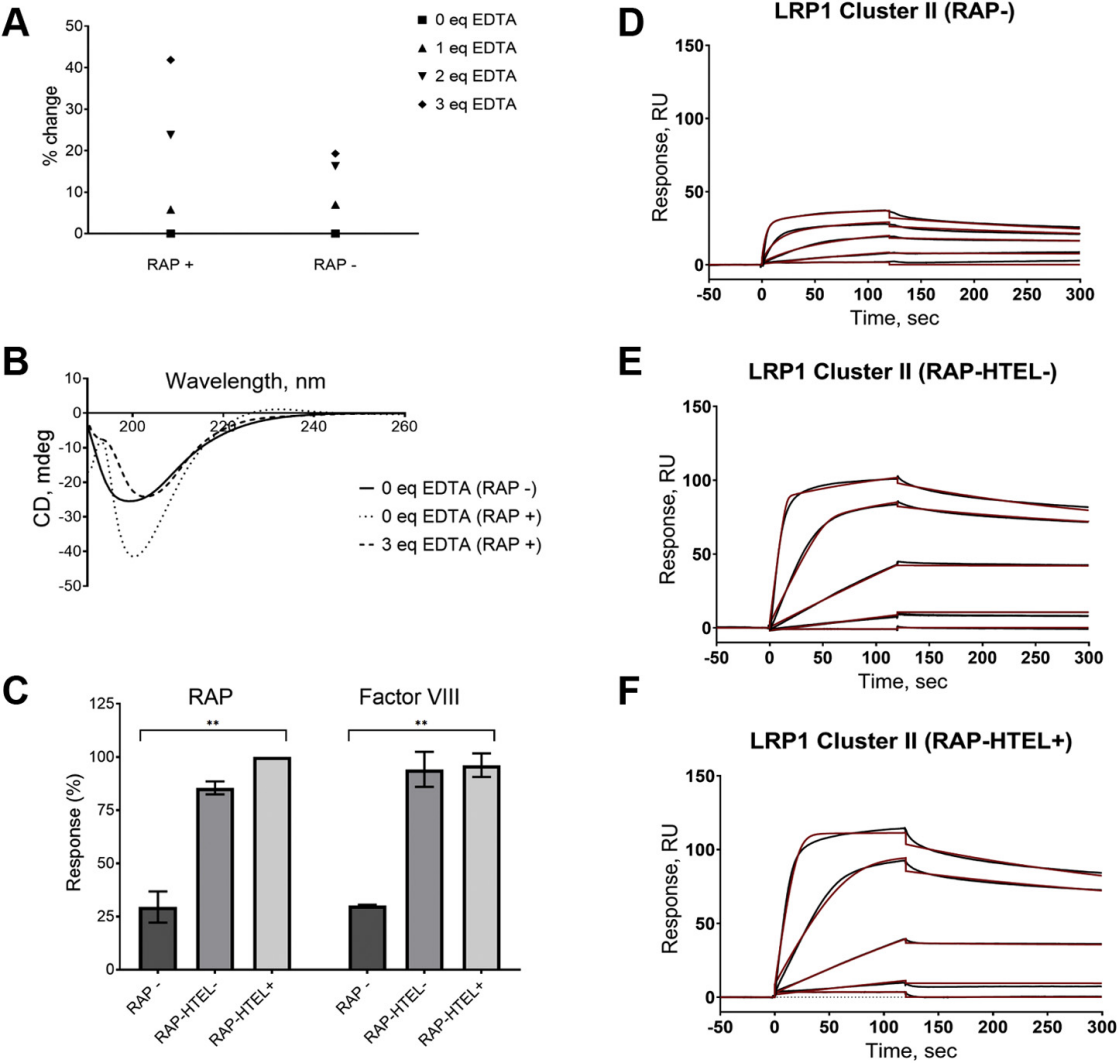
**Coexpression with RAP improves folding and binding properties of LRP1 cluster II.***A*, titration by EDTA of LRP1 cluster II coexpressed with RAP-HTEL (RAP+) or without RAP (RAP−) monitored by CD. Molar equivalent of added EDTA relative concentration of calcium in sample (5 mM); “% change” scale corresponds to a decrease of the negative peak at ~200 nm. *B*, CD spectra of equimolar samples of LRP1 cluster II coexpressed with RAP-HTEL (RAP+) or without RAP (RAP−) (more details are provided in Fig. S2). *C*, binding of LRP1 cluster II (monomer) preparations to RAP and FVIII by SPR. Each preparation of LRP1 cluster II was immobilized at ~250 RU and tested for binding with either RAP (0.08 nM–20 nM) or FVIII (0.8–200 nM) in flow phase using Biacore T200 instrument (Experimental procedures). The R_max_ responses were normalized per RU of immobilized protein (RU/RU) and expressed in percent (% ±SD) relatively the highest signals in each RAP and FVIII group as an average of two independent experiments: RAP−, LRP1 cluster II expressed without RAP (*dark gray*); RAP-HTEL−, LRP1 cluster II coexpressed with RAP not having the HTEL signal (*medium gray*); RAP-HTEL+, LRP1 cluster II coexpressed with RAP having the HTEL signal (*light gray*). *D–F*, *black lines*: real-time binding curves of RAP and immobilized CR fragments in the experiment shown in panel *C*. *Red lines*: fitting the signals using the “bivalent analyte” model, which produced similar results of the steady-state affinity model. The determined K_D_s using the latter model were 1.67 nM for cluster II (RAP−), 1.97 nM for cluster II (RAP-HTEL−), and 2.66 nM for cluster II (RAP-HTEL+). The primary data for FVIII-binding with the LRP1 cluster II variants are available upon request. RAP: *p* value = 0.0012 (∗∗), FVIII: *p* value = 0.0023 (∗∗).

We further tested titration of the protein variants by EDTA to evaluate whether removal of Ca^2+^ from the protein may further define the folding difference and shed some light on possible correlation between the changes in the CD spectra, and content of negatively charged amino acid residues as Ca^2+^ is known to form complexes with negatively charged carboxylate groups of proteins (37, 38). Although it is still unclear whether Ca^2+^ helps the protein to fold or whether Ca^2+^ is incorporated into the proteins after its folding (39), in further assessments, we aimed at the CR-based evaluation of two aspects: (i) whether a coexpression with RAP results in higher amount of protein-bound Ca^2+^ to be assessed by EDTA titration and (ii) whether there is a possible correlation between the percentage of the changes in the CD intensity at the major extremum around 200 nm per an addition of EDTA (% change) and a number of negatively charged amino acid residues in protein (mainly D and E). Consequently, upon titration with EDTA of cluster II variants, we observed more significant change of the CD spectra intensity at 200 nm for protein coexpressed with RAP than that for the counterpart protein (Fig. 3, *A* and *B*; Fig. S2, A–C and H–J), indicating higher content of Ca^2+^ in the RAP coexpressed protein. In further experiments, we also monitored possible correlation between the number of negatively charged amino acid residues in the proteins and level of the EDTA titration-related CD changes of the spectra.

In addition, LRP1 cluster II coexpressed with RAP exhibited a small but well-defined band at 230 nm that disappeared upon addition of EDTA, whereas cluster II expressed without RAP did not exhibit such a band. These differences most likely reflect certain folding/conformational differences with regard to mutual disposition of the tryptophan residues between the protein variants. According to the literature, the small band at 230 nm can be attributed to the presence of tryptophan residues (40, 41, 42, 43) and/or to the presence of disulfide bonds (41, 44, 45). Therefore, we further analyzed our CD data from a standpoint of the presence of tryptophan and cysteine residues (Table 1).

Functional properties of expressed proteins were compared by testing their binding to RAP (commercial) and FVIII, a known ligand of LRP1 (31, 35, 46) using SPR. Immobilized LRP1 cluster II variants, coexpressed with RAP, showed significantly higher binding with RAP and FVIII, than LRP1 cluster II expressed without RAP (Fig. 3*C*) indicating higher content of functional protein in both RAP coexpressed protein variants. The concentration-dependent binding and dissociation signals of RAP and FVIII were fitted with “bivalent analyte” and steady-state affinity models, which resulted in similar affinities. Therefore, the steady-state model producing single value of K_D_ was used. The calculated K_D_s for all three cluster II variants were similar for each of the ligands. For PAP-binding, the K_D_s were in the range of 1–3 nM (Fig. 3, *D*–*F*), which is in agreement to that previously determined for RAP and LRP1 (K_D_ 1–5 nM, (23, 27, 28, 29)). The K_D_s for FVIII binding to LRP1 cluster II coexpressed with RAP, having or not having the HTEL-tag, were 37 nM and 25 nM, respectively consistent with previous results (35, 46), whereas for the binding to LRP1 cluster II, expressed without RAP or commercially acquired, were 244 nM and 450 nM, respectively (data available upon request) indicating worse quality of these LRP1 cluster II preparations.

Thus, both SPR and CD data show that RAP facilitates folding and functional properties of LRP1 cluster II and supports suitability of the chosen methodology for testing interactions of RAP with LDLR family receptors.

### Coexpression with RAP increases yields and improves binding properties of LRP1 clusters II–IV

**Figure 4:**
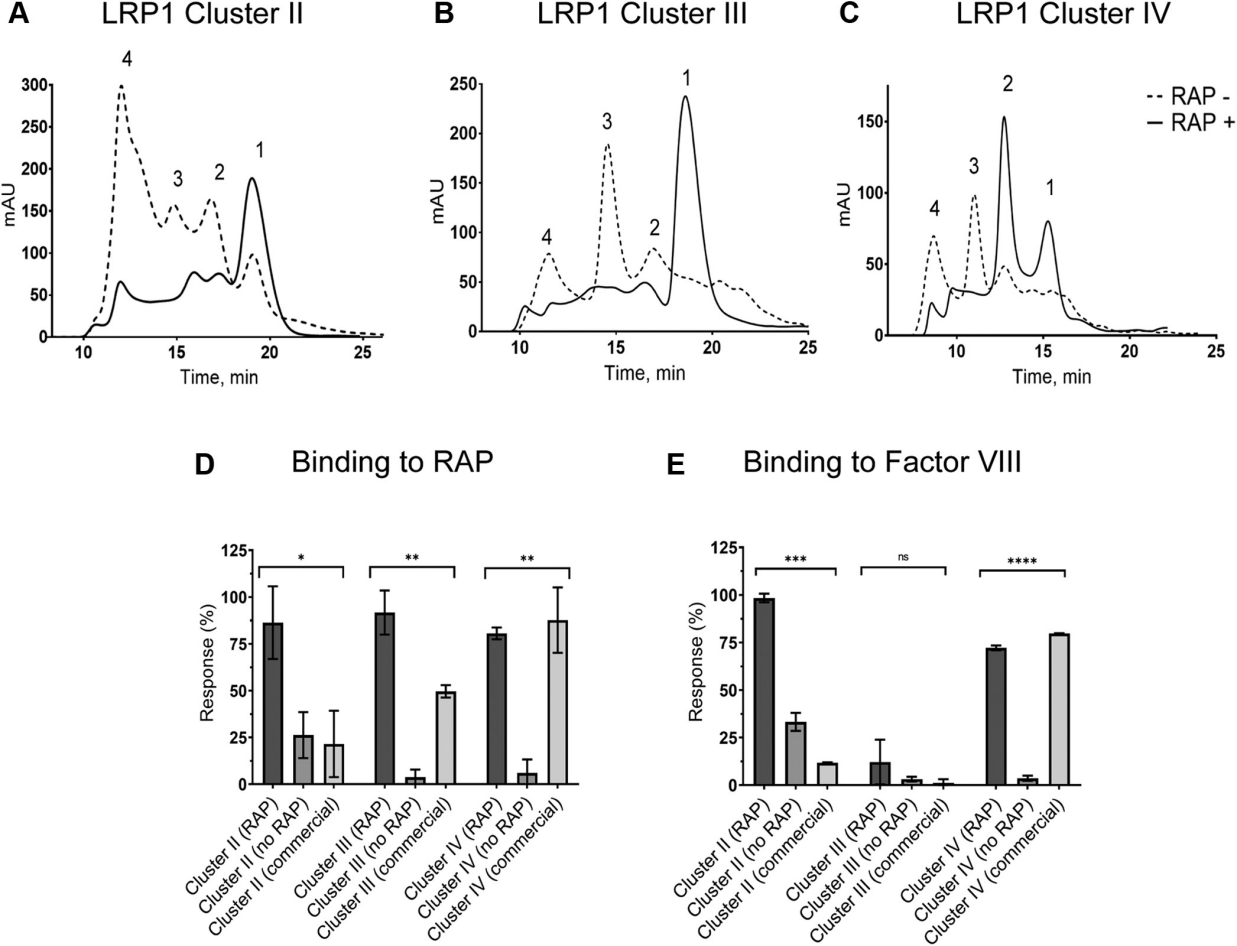
**Coexpression with RAP increases the yields and improves binding properties of LRP1 clusters II, III, and IV.***A–C*, molecular weight profiles of the expressed LRP1 clusters II, III, and IV by SE-FPLC. *Dotted line*: an LRP1 cluster expressed without RAP (RAP−) and purified on a Ni-column using a standard medium-salt/imidazole column wash buffer (Experimental procedures). *Solid line*: an LRP1 cluster coexpressed with RAP (RAP+) and purified on the Ni-column using a high salt/imidazole-EDTA column wash buffer. Such wash was found to remove RAP, tightly bound with the cluster's forms, and the clusters' multimeric forms, whereas the monomeric forms remained bound to the column. The peaks numbering corresponds to 1—monomers (correctly folded protein) and 2, 3, and 4—dimers, trimers, and multimers, respectively (randomly folded protein). *D* and *E*, binding of purified LRP1 clusters II, III, and IV (monomers) to RAP and FVIII by SPR. Each cluster variant, expressed with RAP (*dark gray*), without RAP (*medium gray*), or commercially acquired (*light gray*), was immobilized at ~250 RU and tested for binding with 20 nM of RAP (0.08 nM–20 nM) or FVIII (0.8–200 nM) in flow phase using Biacore T200 instrument (Experimental procedures); the primary data for RAP binding are shown in Figure S3, and for FVIII binding are available upon request. The responses (R_max_) were normalized per RU of immobilized protein (RU/RU) and expressed in percent (% ±SD) relatively the highest signals in each RAP and FVIII groups, as an average of two independent experiments. Binding with RAP: LRP1 cluster II: *p* = 0.0001 (∗), LRP1 cluster III: *p* = 0.0031 (∗∗) and LRP1 cluster IV: *p* = 0.0089 (∗∗); binding with FVIII: LRP1 cluster II: *p* = 0.0002 (∗∗∗), LRP1 cluster III: *p* = 0.3636 (ns), LRP1 cluster IV: *p* < 0.0001 (∗∗∗∗).

Using the same protocol, we generated LRP1 clusters II–IV expressed in the presence or absence of RAP (without HTEL) in scaled-up conditions (1 L of expression media). For each protein, the SE-FPLC profile demonstrated several-fold increase of the monomer level upon coexpression with RAP (Fig. 4, *A*–*C*). Compared with conditions of protein purification in initial experiments, the data show that use of high salt/imidazole/EDTA buffer for the Ni-column wash resulted in removal of the protein multimeric forms compared with the use of standard medium salt/imidazole buffer. Notably, the multimeric forms also contained coexpressed RAP (solid lines) based on shifts of respective elution peaks for protein expressed without RAP (dotted lines). The protein yields are shown in Table S2.

By SPR analysis, purified LRP1 clusters II–IV coexpressed with RAP demonstrated higher binding signals with both RAP (commercial) and FVIII compared with proteins expressed without RAP or to control commercial LRP1 clusters II–IV (Fig. 3, *D*–*F*, Fig. 4, *D* and *E*, and Fig. S3, *A*, *D*, and *G*). For binding RAP, the proteins coexpressed with RAP or not had similar affinities with respective K_D_s: (i) 2.4 ± 0.3 nM and 1.2 ± 0.7 nM for LRP1 cluster II, (ii) 2.0 ± 0.6 nM for LRP1 cluster III coexpressed with RAP, and (iii) 1.4 ± 3.9 nM for LRP1 cluster IV coexpressed with RAP (no binding of clusters III and IV expressed without RAP was observed); representative experiments are shown in Figure 3, *D*–*F* and Figure S3, *B–F*. All these K_D_s values are similar to the K_D_ for RAP and LRP1 interaction (K_D_ 1–5 nM, (23, 27, 28, 29)). FVIII showed binding to LRP1 cluster II expressed with or without RAP (K_D_s 42.0 ± 0.7 nM and 191.5 ± 74.2 nM) and to LRP1 cluster IV expressed with RAP (K_D_s 10.3 ± 3.0 nM and 61.9 ± 10.0 nM) or commercial LRP1 cluster II, whereas no binding to any preparation of LRP1 cluster III was observed (Fig. 4*E*). Notably, (i) the commercial LRP1 cluster II had significantly lower binding to both RAP and FVIII, and (ii) the ability of FVIII to bind with LRP1 clusters II and IV, but not LRP1 cluster III, is consistent with previous results (35, 46). Thus, coexpression with RAP improved yield, folding, and ligand-binding properties of LRP1 clusters II–IV in accordance with the known role of RAP as folding chaperone for LRP1.

### Coexpression with RAP increases yield and improves properties of LDLR and vLDLR CR clusters

**Figure 5:**
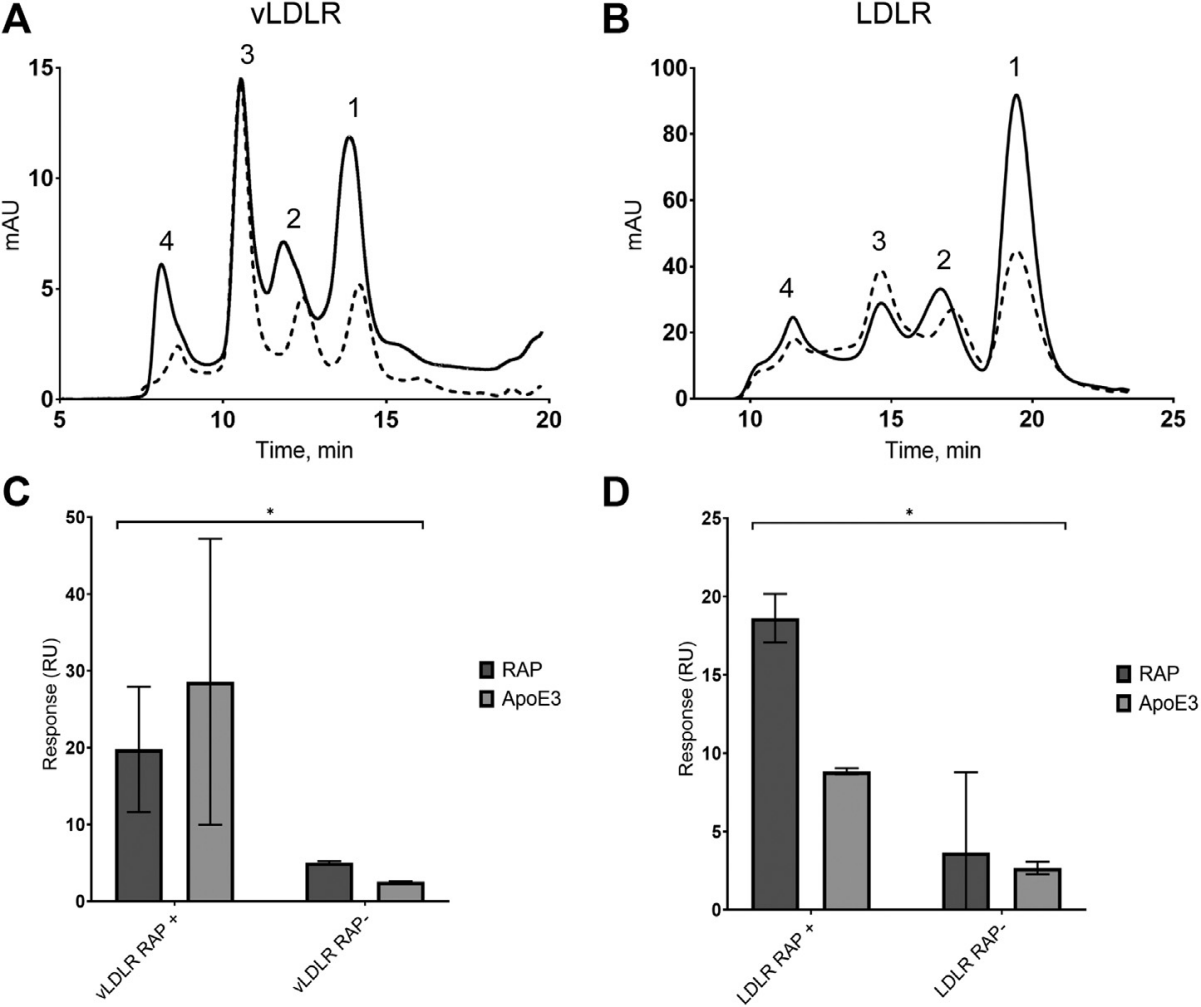
**Coexpression with RAP increases yield and improves binding properties of LDLR and vLDLR CR clusters.** Molecular weight profiles of expressed vLDLR (*A*) and LDLR (*B*) CR-clusters by SE-FPLC. *Dotted line*: protein expressed without RAP (RAP−); *solid line*: protein coexpressed with RAP (RAP+). The peaks numbering corresponds to: 1—monomers (correctly folded protein), and 2, 3, and 4—dimers, trimers, and multimers, respectively (randomly folded protein). Binding of purified and immobilized at ~250 RU vLDLR (*C*) and LDLR (*D*) monomers with of RAP (0.08 nM–20 nM) (*dark gray*) and 200 nM of ApoE3 (0.78 nM–200 nM) (*gray*) in flow phase by SPR using Biacore T200 instrument (Experimental procedures); the primary data are shown in Figures S3 and S4. The responses (R_max_) were normalized per RU of immobilized protein and expressed as averages of two independent experiments (RU/RU ± SD). Two-way ANOVA: vLDLR *p* value = 0.0318 (∗); LDLR *p* value = 0.0477 (∗).

Based on comparable sizes of the CR clusters of LDLR and vLDLR, and LRP1 clusters II-IV (Fig. 1*B*), we expressed the clusters of LDLR and vLDLR with and without RAP. The SE-FPLC profiles of the proteins coexpressed with RAP demonstrated an increase in 2–2.5-fold of each monomer levels shown in a representative experiment in Figure 5, *A* and *B* and increased purification yields (Table S2). According to CD data, both proteins expressed with RAP had better ordered structures compared with their respective counterparts expressed without RAP (Table 1). vLDLR cluster expressed with RAP had lower percentage of unordered structure elements and presence of the peak at ∼230 nm indicating difference in the structure. These results indicated better folding of the both proteins expressed with RAP.

By SPR analysis, the proteins expressed with RAP demonstrated significantly higher binding with RAP and ApoE3, a ligand of both LDLR and vLDLR (47, 48), compared with proteins expressed without RAP (Fig. 5, *C* and *D*); representative experiments are shown in Figures S3, *J–M* and S4. Notably, (i) no binding of RAP to LDLR cluster expressed without RAP was observed and (ii) the affinity of ApoE3 to vLDLR cluster (K_D_ ∼17 nM) was higher than to LDLR cluster (K_D_ ∼76 nM) (Fig. S4). In contrast to LRP1 clusters II–IV, both proteins expressed without RAP showed more significant reduction in binding both ligands indicating lower ability of the CR moiety in LDLR and vLDLR to self-fold compared with LRP1. Altogether, these data are in accordance with the known role of RAP of folding chaperone of vLDLR and indicate such role for LDLR.

### RAP binds to the majority of CR doublets within clusters II and IV of LRP1

**Figure 6:**
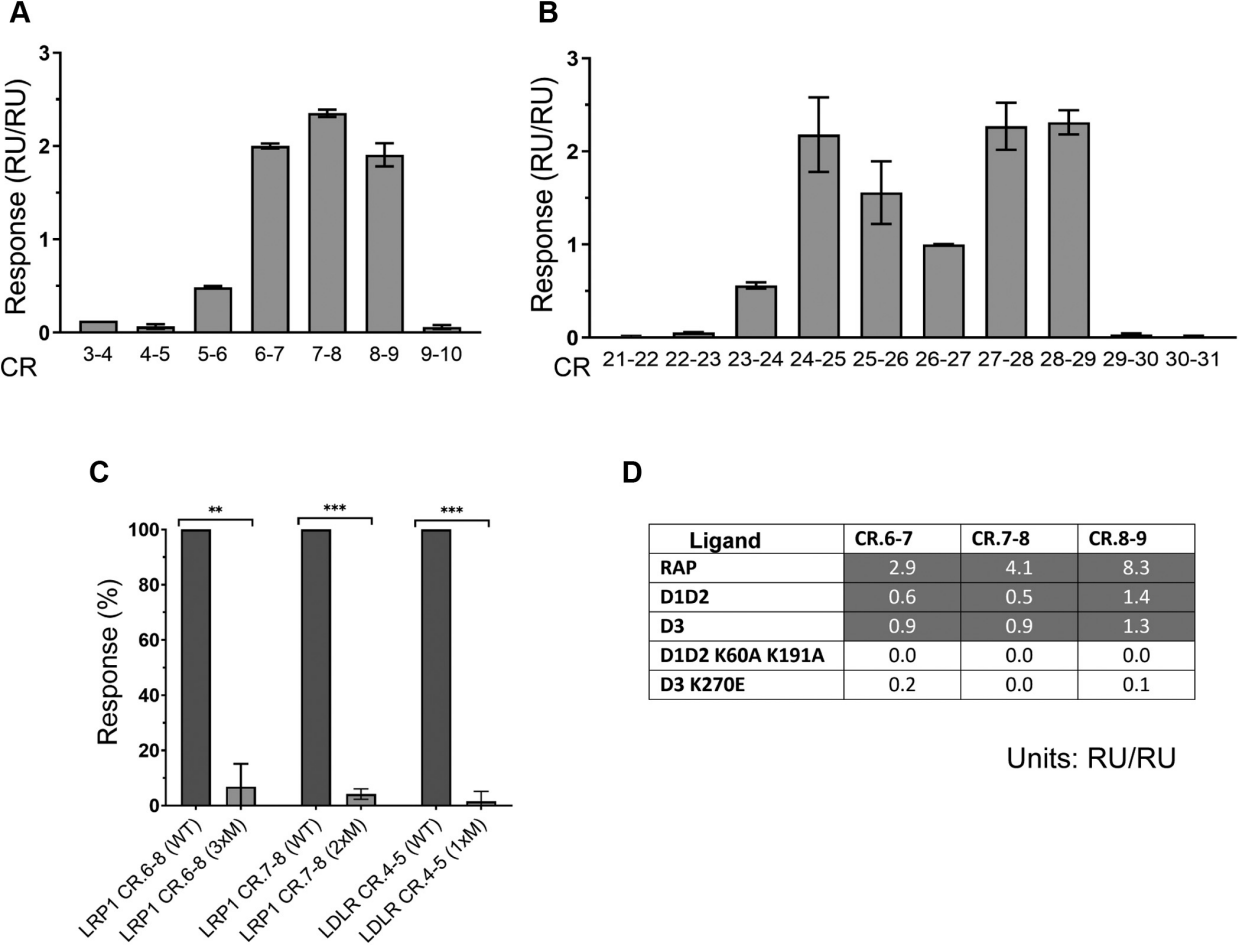
**Characterization of bivalent sites for binding RAP in LRP1 clusters II and IV by SPR.***A* and *B*, binding RAP to recombinant CR doublets overlapping LRP1 clusters II (CR.3–10) (*A*) and IV (CR.21–31) (*B*). Each CR doublet was immobilized at ~1000 RU and tested for binding with 10 nM of RAP using Biacore 3000 instrument (Experimental procedures). The responses were normalized per RU of the CR-doublet immobilization level (RU/RU) multiplied by 10 for convenience of data visualization and further normalized to the average of the two independent experimental data (±SD) to adjust scales. *C*, binding of RAP to selected high-binder CR-fragments (WT, wild-type) including those with mutations (M) of the conserved tryptophans: LRP1 CR.6–8 (3xM; W994S; W1032S, W1080S), LRP1 CR.7–8 (2xM; W1032S, W1080S), and (additional controls) LDLR CR.4–5 (WT) and its mutant (1xM, W214S) in conditions as those in panels *A* and *B*. The responses were normalized per RU of immobilized protein (RU/RU) and expressed as percent (% ± SD) relatively signals produced by respective nonmutated (WT) fragments. *D*, binding of RAP D1/D2 and D3 fragments with mutations of “critical” lysines to LRP1 CR-doublets 6–7, 7–8, and 8–9. The proteins were immobilized and tested for binding with RAP, its recombinant D1/D2 and D3 domains, and their variants with mutations of “critical” lysines: D1/D2 K60A/K191A and D3 K270E (Experimental procedures). Data shown as average response normalized to protein immobilization level (RU/RU), multiplied by 10 for convenience of data visualization. In gray highlighted data for which ligand binding was observed. LRP1 CR.6–8 *p* value = 0.0040 (∗∗); LRP1 CR.7–8 *p* value = 0.0002 (∗∗∗); LDLR CR.4–5 *p* value = 0.0007 (∗∗∗).

To get a deeper insight on the mechanism of interaction of RAP and LRP1, we mapped minimal sites of LRP1 clusters II and IV providing bivalent interactions with RAP to supplement such data obtained by us previously for LRP1 cluster III (28). This was performed upon generation of CR doublets overlapping both clusters and testing them for binding to RAP similar to that performed for LRP1 cluster III (28). Most of the CR doublets interacted with RAP with highest signals for those overlapping the regions CR.5–9 of LRP1 cluster II and CR.23–29 of LRP1 cluster IV (Fig. 6, *A* and *B*). Comparing these results with those for LRP1 cluster III (the core binding region is CR.15–19) shows that all CR doublets of LRP1 with strong binding to RAP contain the conserved tryptophan in both CR domains, while the doublets with weaker binding contain less conserved phenylalanine at homologous position(s) (Fig. S1). In turn, the absence of the aromatic residue in one domain of CR.9–10 and CR.29–30 correlates with their inability to bind RAP. Respectively, the absence of such aromatic residue in both CR domains of CR.30–31, and also CR.1–2 (46), correlates with entire inability to bind RAP. These observations are in accordance with data of *Fisher et al.* (15) showing criticality of the aromatic residues within a CR doublet of LDLR for interaction with RAP.

### Mutational analysis of RAP D1/D2 and D3 interactions with CR-fragments of LRP1 supports criticality of specific amino acid residues and the bivalent mode of these interactions

Previous studies demonstrated criticality of specific lysines pairs within each D1/D2 and D3 of RAP for their binding to LRP1 (23, 49). This indicates that the same lysines are critical for RAP binding to any of binding active CR doublet of LRP1. This was verified by testing isolated D1/D2 and D3 with mutations of the “critical” lysines with selected CR fragments of LRP1. We also used the CR fragments with mutations of the conserved tryptophans to verify their importance for the interactions. Thus, we used LRP1 CR doublets 6–7, 7–8, 8–9, CR triplet 6–8, and some of these fragments with W→S mutations at W994 (CR.6), W1032 (CR.7), W1080 (CR.8), W3629S (CR.28), and W3670 (CR.29) (Fig. S1); previously we showed that such mutation does not affect the CR domain structure (32). We also used LDLR CR.4–5 and its W214S variant. The fragments were tested with full-length RAP, its isolated D1/D2 and D3 fragments, and the fragments' variants with mutations of “critical” lysines K60 and K191 in D1/D2 (23) and K270 in D3 (50).

By SPR analysis, all CR fragments bound RAP and both of its D1/D2 and D3 with similar signals and affinities (Fig. 6, *C* and *D*) comparable with affinities of RAP for isolated clusters II–IV of LRP1 (Fig. S3). However, all CR fragments were unable to bind mutated RAP fragments, D1/K60A/D2/K191A and D3/K270E (Fig. 6*C*). Similarly, the CR fragments W→S variants were unable to bind RAP, D1/D2, and D3, or had significant decrease in binding. Importantly, mutating only one of the lysines in RAP D3 (K270E) or only one of the tryptophans in a CR doublet (LDLR CR.4–5 W214S) resulted in abolishment of the binding, confirming criticality of the mutated residues, and consistent with such effect observed by us previously for LDLR CR.4–5 W165S/W214S (32). Notably, the triplet LRP1 CR.6–8 binding to RAP was similar to doublets LRP1 CR.6–7 and LRP1 CR.7–8 indicating that only one of those within the triplet is available for binding with RAP at a given time point. Altogether, these results show that a pair of the “critical” lysines on either of D1/D2 or D3 portions of RAP and a pair of conserved tryptophans within a CR doublet are critical to support the bivalent interaction. This indicates that during RAP and LRP1 interaction, any bivalent combination being formed between the molecules involves either pair of “critical” lysines K60A/K191 or K256/K270 on the RAP side and the both conserved aromatic residues within the interacting CR doublet on the receptor side.

### Coexpression with RAP improves folding of small CR fragments

Next, we aimed to verify if the bivalent mode previously described for interaction of RAP D1/D2 and LRP1 in purified system (23) is applicable to the environment within living cell. This was tested by coexpression of RAP with its most binding-active CR-doublets 6–7 and 7–8 of LRP1 cluster II (Fig. 6*A*). Notably, the linkers connecting the domains in both doublets cover extremes in length among all CR linkers of LRP1: while CR.6–7 has the shortest linker formed by three amino acids, CR.7–8 has the longest linker formed by ten amino acids (Fig. S1). We also tested RAP coexpression with control fragments: triplet CR.6–8 comprising both doublets, singlet CR.7, a part of each doublet, and CR.30–31, a unique doublet, which does not have the conserved tryptophans in both domains and is not able to interact with RAP (Fig. 6*B*).

**Figure 7:**
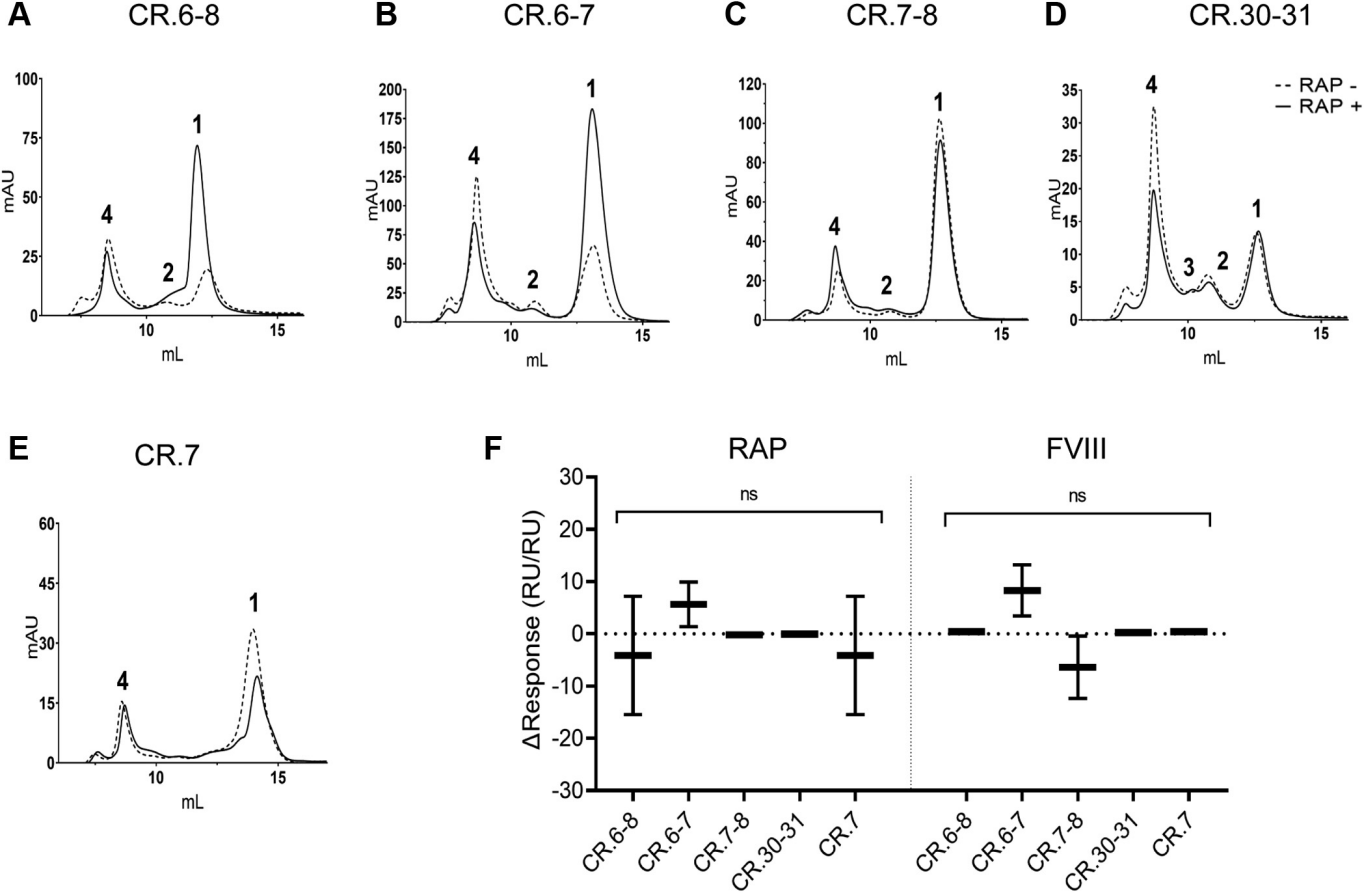
**Testing of molecular forms and binding properties of selected CR doublets of LRP1 upon coexpression with RAP.***A*–*C*, SE-FPLC molecular weight profiles of the expressed CR fragments expressed with or without RAP: CR.6–8 (*A*), CR.6–7 (*B*), CR.–8 (*C*), CR.30–31 (*D*), and CR.7 (*E*) *dotted line*: protein expressed without RAP (RAP−), *solid line*: protein coexpressed with RAP (RAP+). The peaks numbering corresponds to: 1—monomers (correctly folded protein), 2, 3, and 4—dimers, trimers, and multimers, respectively (randomly folded protein). *F*, difference in response of CR fragments expressed with and without RAP upon binding to RAP or FVIII by SPR. The CR fragments were immobilized at ~250 RU and tested for binding with RAP (0.08 nM–20 nM, *dark gray*) and FVIII (0.8 nM–200 nM, *gray*) using Biacore T200 instrument. ΔResponse was calculated as difference in response between samples coexpressed with RAP and samples expressed without RAP; plot: min to max. The Response (RU/RU) was calculated as a normalized response (R_max_) to immobilization level and expressed as the average of two independent experiments (RU/RU ± SD). The primary data for RAP are shown in Figure S5 and for FVIII are available upon request. One-way ANOVA comparison between CR domains within the group (RAP or FVIII): not significant (ns).

Upon coexpression with RAP, we observed a 3–4-fold increase of both CR.6–8 and CR.6–7 monomer peaks by SE-FPLC, while the monomer peaks of other CR-fragments were not increased (Fig. 7, *A*–*E*). This indicates that during protein coexpression, RAP interacted with both CR.6–7 and CR.6–8 to facilitate their folding, whereas it did not interact with CR.7–8 despite its ability to bind RAP in purified system (Fig. 6*A*). This suggests the increase of expression of CR.6–8 triplet was due to interaction of its CR.6–7 doublet with RAP, but not of CR.7–8. The absence of effect of RAP coexpression on CR.30–31 and CR.7 was expected due to the discussed structural reasons; the even lower level of the CR.7 monomer upon RAP coexpression was attributed to higher burden on the cells' expression machinery by RAP coexpression. Resulting purification yields were proportional to respective monomer levels (Table S2).

According to CD evaluation, the presence of the 230 nm band correlates with the number of tryptophan residues: if the number of those in a protein is greater than 1, a well-developed 230 nm band can be observed, as seen only for the protein coexpressed with RAP; whereas, for proteins with only one tryptophan, like CR 7 and CR 30–31, no CD was observed at 230 nm (Fig. S2, *F* and *M*; Table 1). At the same time, the spectra of CR.30–31 having a single tryptophan (nonconserved), both variants did not exhibit any band at ∼230 nm, in contrast to CR.7–8, which exhibited the ∼230 nm band only when coexpressed with RAP (Table 1). Thus, based on the relatively small difference between CD spectra (including EDTA titrations) and secondary structure determinations, it seems that RAP did not significantly participate in the folding of CR.30–31 and CR.7 during expression, while it interacted intracellularly with CR.6–8, CR.6–7, and even with CR.7–8 due to the differences in folding of these proteins coexpressed with RAP compared with those expressed without RAP. Another conclusion that can be drawn upon the testing of all expressed CR fragments in this study is that we did not find a direct correlation between the number of negatively charged amino acid residues in tested proteins and the level of respective EDTA-related CD changes.

By SPR analysis, we observed no significant difference between proteins expressed with or without RAP in binding to RAP or FVIII (Fig. 7*F*). Both preparations of CR.30–31 did not interact with RAP and FVIII as expected, whereas other CR fragments had similar signal levels and affinities in the binding whether they were coexpressed or not with RAP (Fig. S5, *G–H*). For binding RAP, the respective K_D_s were: (i) 2.8 ± 1.9 nM and 2.9 ± 1.0 nM for CR.6–8; (ii) 3.8 ± 1.5 nM and 3.9 ± 1.7 nM for CR.6–7; (iii) 3.1 ± 3.1 nM for CR.7–8; 34.9 ± 5.3 nM and 43.7 ± 34.2 nM for CR.7, shown in a representative experiment in Figure S5. Notably, 10–20 times increase in K_D_ for CR.7 compared with those of CR.6–7 and CR.7–8 (comprising CR.7) reflects a contribution of a monovalent binding combination into the bivalent combination. This decrease in affinity of CR.7 is consistent with 50–100 times increase in K_D_s when isolated RAP D1 and D2 domains, bearing single “critical” lysines, interacted with LRP1 (23) most likely in the monovalent mode. The difference in binding of FVIII to CR fragments coexpressed with RAP or not is shown in Figure 7*F*; no binding was found to CR.30–31 consistent with the absence of the conserved tryptophans and to CR.7 consistent with its assumed weaker potential to bind ligands *via* monovalent interaction.

Altogether, the results show that RAP improves folding of the model CR doublets upon coexpression and confirm relevance of their bivalent interaction mode to the environment within living cell. Furthermore, similarity of affinities of RAP to CR doublets 6–7 and 7–8, CR triplet CR.6–8, and CR clusters of LRP1 being in low nanomolar range indicates equivalency of these interactions, *i.e.*, bivalent mode of those.

### *In silico* docking of RAP and CR.6–8 triplet indicates formation of alternative bivalent combinations

**Figure 8:**
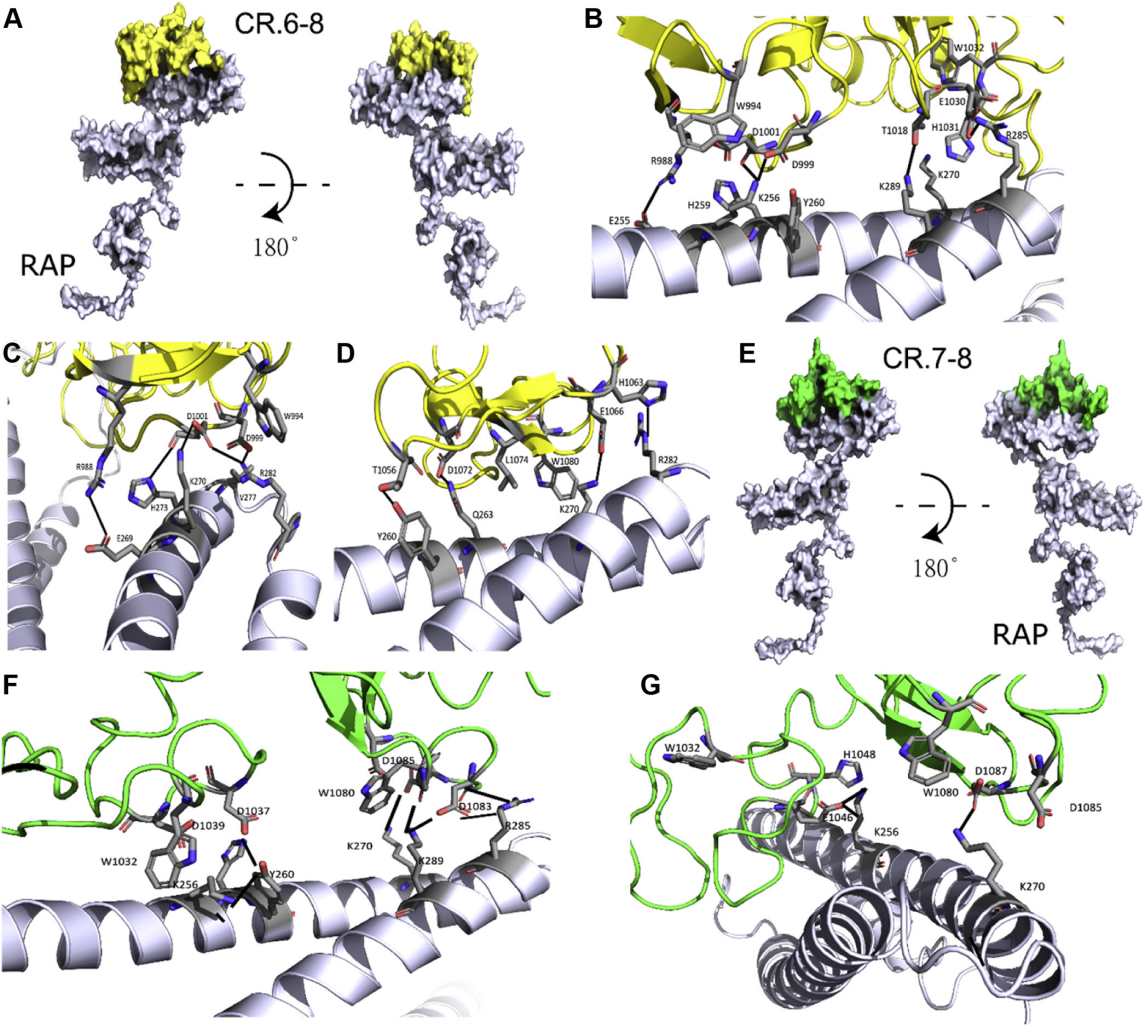
**Modeling of CR domains interaction with RAP.***A*, the top-scoring interface energy model of LRP1 CR.6–8 triplet complexed with RAP is shown from two angles. The LRP1 CR.6–8 is colored in *yellow* while RAP is colored in *blue white*. A closer look at the top scoring models showed two binding modes: bidentate (*B*) and single (*C* and *D*). *E*, the top-scoring LRP1 CR.7–8 doublet model complexed with RAP is shown from two angles; the LRP1 CR.7–8 doublet is colored in *green* while RAP is colored in *blue white*. A closer look at the top scoring models also showed two binding modes: bidentate (*F*) and single (*G*). The structure of RAP was taken from the Protein Data Bank (PDB 2P01), and the structures of LRP1 CR.6–8 and LRP1 CR.7–8 were built using the I-TASSER server.

To obtain an insight on the relationship of overlapping CR doublets during interaction of RAP and LRP1, we performed *in silico* docking of the triplet CR.6–8 to RAP using the RosettaDock program (51, 52, 53, 54, 55). The initial run and its refinement indicated two modes of interactions (Fig. S6) corresponding to single and bidentate orientations of the triplet relatively RAP (Fig. 8); the respective top interface energy scores are shown in Figure S7, *A* and *B*.

These interactions mainly involved docking of CR.6–7 with RAP D3 in a bidentate mode, where each CR domain interacted with the “critical” lysines K256 and K270 (Fig. 8*B*), and also involved electrostatic interactions of the conserved acidic residues (D, E) of the CRs (Fig. S1) D999/D1001 with K256 (Fig. 8*B*), D1001 with K270 (Fig. 8*C*), and E1066 with K270 (Fig. 7*D*), where the conserved tryptophans W994 (CR.6), W1032 (CR.7), and W1080 (CR.8) served as space fillers to create hydrophobic pockets between the molecules allowing other residues in the interface to make favorable interactions (Fig. 8, *B*–*D*). Refinement of these models showed additional bidentate and single binding modes (Fig. S8), where the residues around W1080 interacted with the residues around K253 (Fig. S8*A*), and residues around W994 and W1032 interacted with K256 and K270 (Fig. S8*B*).

As the docking of CR.6–8 mainly captured a bidentate mode for the CR.6–7 interactions, we tested the docking of CR.7–8 separated from the triplet. The top-scoring interface-energy models also identified two binding modes, single and bidentate (Fig. S6, *F* and *G* and Fig. S7, *C* and *D*), where CR.7–8 docked with RAP D3 as shown for one of the models in Figure 8*E*. Figure 8*F* shows a model depicting a bidentate mode, where D3 K256 and K270 form electrostatic interactions with the acidic residues on CRs 7 and 8, respectively, and also, K256 fills space next to W1032 (CR.7) and K270 forms interactions near W1080 (CR.8); Figure 8*G* shows a model depicting a single interaction, where K270 occupies space next to W1080.

Notably, the dominant role of CR.6–7 over that of CR.7–8 in docking of CR.6–8 with RAP is consistent with the preferable interaction of RAP with CR.6–7 over CR.7–8 upon coexpression. We propose that the binding prevalence of CR.6–7 observed in both experimental setups was due to significantly shorter interdomain linker within CR.6–7 providing higher number of the conformations favorable for the binding.

Overall, RAP D3 lysines 256 and 270 were able to dock any domain of the CR.6–8 triplet, where they played a key role. In particular, (i) K256 formed favorable binding combinations with CR.6 (Fig. 8*B*), CR.7 (Fig. 8*F*), and CR.8 (Fig. S8*A*), and (ii) K270 formed favorable combinations also with CR.6 (Fig. 8*C*), CR.7 (Fig. 8*B*), and CR.8 (Fig. 8, *F* and *G*). During these interactions, each of CR.6–7 and CR.7–8 formed alternative bivalent combinations with either lysine of RAP. These data are consistent with such role of K256 and K270 on D3 and the conserved residues (W, D) within the complexes of RAP D3 with LRP1 CR.5–6 (26) and LDLR CR.3–4 (15), and support a universal role of these residues in interactions of RAP and LDLR family receptors. Collectively, the results of our study indicate that RAP and LRP1 interact *via* the formation of multiple bivalent combinations being switched over time in a dynamic mode.

## Discussion

In this study, we investigated mechanisms of interaction of RAP with selected LDLR family receptors mainly focusing on the interaction with LRP1. In an intracellular environment, coexpressed RAP was found to interact with isolated CR clusters of LRP1, vLDLR, and LDLR based on improvement of protein yields, folding, and ligand-binding properties. Folding of proteins expressed with and without RAP was assessed upon comparison of percentage of the secondary structure elements, content of unordered fraction, and spectra upon titration with EDTA by CD. For the tested CR clusters of receptors, RAP coexpressed variants had generally stronger negative extremum at ∼200 nm, indicative for higher content of β-structures, and a small but well-defined band at ∼230 nm compared with proteins expressed without RAP. Upon titration with EDTA, the proteins coexpressed with RAP exhibited disappearance of the band at ∼230 nm and a more significant change of the CD intensity at ∼200 nm than the counterpart proteins, which indicate that the ordered CR domains better retain Ca^2+^ (Table 1). These results are consistent with general improvement of binding properties of proteins coexpressed with RAP by SPR in our study, and data of a previous study showed that higher content of Ca^2+^ in LDLR favored its binding properties (56). Thus, coexpression of the proteins with RAP improved their yields and properties.

Results of our study support the role of RAP as molecular chaperone for the tested receptors and are in accordance with such conclusions for LRP1 and vLDLR shown previously (20, 21, 22). However, the chaperone function of RAP for LDLR was not supported in previous studies most likely due to the difference in experimental conditions: two studies used a mouse model with knockout of RAP gene (21, 22), and one study used a cell culture model with cotransfection of separate constructs coding for RAP and a receptor (20). In contrast, we used a single construct with both genes to ensure proteins' coexpression within the same cell. A limitation of our approach is that in these conditions, overexpression of both proteins may exaggerate their interactions *in vivo*. In particular, RAP was cosecreted in a complex with CR fragments, despite having an insect ER recycling signal (HTEL), most likely due to oversaturation of the cells' machinery to recycle the protein. However, we considered such effect beneficial for preserving the CR fragments quality until removal of RAP upon protein purification. Despite our model's limitation, the chaperone role of RAP for LDLR is consistent with their ability to interact in a purified system (19, 32, 34). Notably, real-time binding by SPR showed similarity of RAP and LDLR affinity (K_D_ ∼2 nM) (32), while an ELISA- and cell-culture-based study showed their affinity to be significantly less (K_D_ 50–250 nM) (34).

In next part of the study, we dissected the complex interaction of RAP and LRP1 into simpler bivalent interactions to characterize the most critical elements of both molecules. To assess all bivalent elements in LRP1, we determined its most binding active CR doublets toward RAP in clusters II and IV, which supplemented such results obtained by us previously for cluster III (28). Together with our previous mapping of the bivalent sites in LDLR (32), this showed that the most RAP-binding active CR doublets of the both receptors in each CR singlet have conserved tryptophan, homologous to W1032 in CR.7 of LRP1, with less frequency of phenylalanine at this position, which is consistent with previous studies, which involved different LDLR family receptors and ligands (13, 14, 15, 26). In turn, the absence of the conserved aromatic residue in any of domains of CR doublet correlates with its inability to bind RAP. Thus, the bivalent sites for binding RAP in LRP1 and LDLR are presented by multiple overlapping CR doublets containing a conserved aromatic residue, preferably tryptophan, in each CR domain.

Relevance of these interactions to *in vivo* conditions was supported by the increased yield of a model CR doublet 6–7 upon its coexpression with RAP. This indicated that RAP directly interacted with the CR.6–7 within cell and assisted its folding. Notably, this doublet is similar to majority of those in LRP1 by the length of interdomain linker composed of three peptide bonds. In contrast, we did not observe an increase of the yield of the doublet CR.7–8 upon coexpression with RAP. This was attributed to uniquely long linker connecting the domains, comprised of ten polypeptide bonds and thus allowing for significantly lower number of the doublet's conformations favorable to match the lysines on RAP. Notably, the yield of a control CR.30–31 doublet was also not improved upon coexpression with RAP, which is consistent with the absence of the conserved aromatic residues in each domain of the doublet and its inability to bind RAP by SPR. Altogether, the results indicate that during biosynthesis of LRP1, RAP interacts with CR doublets of the receptor and facilitates their folding, while this may not be relevant to all CR doublets. However, the ability of such doublets to interact with RAP *in vitro* may reflect another function of RAP—to bind the CR moiety and protect it from premature binding to other ligands during biosynthesis.

The mutational analysis of RAP and LRP1 fragments' interactions indicated that the most critical elements of each molecule for formation of multiple bivalent combinations are essentially the same. This was demonstrated using RAP fragments D1/D2 and D3 having mutations of the “critical” lysines K60/K191 and K270, respectively (23, 26), which were tested with selected CR fragments of LRP1 (and also LDLR) having mutations of the conserved tryptophans. We found that mutation of any of these residues on either side of the ligands resulted in abolishment or significant decrease of their interaction. In regard to RAP, these data agree with previous studies showing criticality of the above lysines for interactions of RAP D1/D2 and D3 fragments with LRP1 (23, 49), which implies that any CR doublet of LRP1 can interact with any of these pairs of the lysines *via* the mechanism described by *Fisher et al.* (15). This is consistent with results of *Jensen et al*, who showed that isolated RAP D3 can interact with each of CR doublets overlapping LRP1 cluster II (except CR.9–10) with comparable affinities (57). These data indicate that any pair of the “critical” lysines of RAP can form a bivalent interaction with any CR doublet of LRP1 bearing the conserved aromatic residue on each CR domain.

Furthermore, the results of our *in silico* study indicate that a particular CR doublet can form two bivalent combinations with the same pair of “critical” lysines, which can “switch” between the CR domains of the doublet. Indeed docking of the model CR triplet 6–8 to RAP showed formation of four alternative bivalent combinations between either of the doublets 6–7 or 7–8 with either of K256 or K270 of D3 by the mechanism described by *Fisher et al.* (15). This indicates the ability of any “critical” lysine of RAP to interact with essentially all CR domains of the receptor.

Upon the docking of RAP with CR.6–8, preferable binding combinations occurred between RAP D3 and CR.6–7. It was most likely related to higher probability of these fragments to acquire conformations favorable for the interaction: in D3, the “critical” lysines K256 and K270 are located on a rigid helical structure, thus have minimal mutual flexibility compared with the lysines K60 and K191 located on different domains of D1/D2. The preference of CR.6–7 over CR.7–8 due to the difference in length of the inter domain linkers was discussed above. Thus, the results of the *in silico* study are consistent with results of protein expression experiment, which indicated more pronounced interaction of CR.6–7 with RAP in cell environment compared with CR.7–8. Altogether, our results indicate that RAP and LRP1 can form numerous bivalent combinations during the interaction.

This raises a question of how all these binding combinations are coordinated during the molecules' interaction. To address this question, we consider that the affinities of RAP and CR fragments tested in our study, *i.e.*, model CR doublets, the CR triplet, and CR clusters II–IV, were similar and in turn, similar to the affinity of RAP and LRP (23, 27, 28, 29) with all respective K_D_s in low nanomolar range (2–5 nM) showing essential equivalency of all these interactions, *i.e.*, bivalent mode of those. Therefore, we propose that at each moment, only one of two sites of RAP interact with a CR doublet of LRP1 and such combinations are constantly switched. In this dynamic mode, RAP statistically contacts all binding active CR domains over time to perform its chaperon function. In this process, some CR domains may not be assisted by RAP for the folding, anyway, RAP still binds them to prevent their premature interactions with other ligands.

Indeed, if the interaction was more complex, *i.e.*, involved both sites of RAP simultaneously forming two bivalent combinations with LRP1, the tetravalent binding mode of it would result in superhigh affinity in K_D_ in a low femtomolar range (57). Based on this assessment, a hypothetical trivalent mode of such interaction involving three “critical” lysines of RAP and three CRs of LRP1 would result in still unrealistically high affinity with K_D_ in a low picomolar range, an intermediate between the low nanomolar range of K_D_ experimentally observed for the bivalent mode. This assessment is consistent with significant increase in affinity of a bivalent complex of LRP1 and PAI upon addition of the third valency upon formation of a tertiary complex with urokinase-type plasminogen activator (uPA), reflected by 100-times decrease in K_D_ (from ∼74 nM to ∼0.9 nM (36)). Notably, experimental differences in K_D_ per valency for the interacting RAP and LRP1 were observed to be in 10–20 times upon testing the binding properties of an isolated CR domain (CR.7) and respective CR doublets and 50–100-times in the study of *Prasad et al.* (23) upon testing interactions of isolated A1 and A2 domains of RAP with LRP1.

Consistent with the above, the studies of *Jensen et al.* (57) and *Gettins et al.* (58) demonstrated that two adjacent CR domains provide most of the binding energy and contribution of the third CR domain observed in some cases was modest. Such effect was also observed for fibrin and vLDLR interaction where a CR doublet of the receptor had a dominant role in the binding with moderate contribution of an adjacent third CR domain (59). We propose that in such cases, the third CR domain may form an additional weaker electrostatic interaction with RAP *via* noncanonical mechanism. In our study, we did not observe such an effect upon testing interactions of RAP and a CR triplet from LRP1 cluster II and both of its overlapping CR doublets, as respective affinities were comparable.

The suggested “dynamic bivalent” mechanism of RAP and LRP1 interaction is in accordance with data previously observed for RAP and LDLR interaction, where multiple CR doublets of LDLR were found to interact with RAP with affinities similar to those of the CR cluster and exodomain of the receptor (32). Therefore, we propose that this mode may be relevant to also interactions of RAP with LDLR, and furthermore with all receptors-members of the LDLR family. Notably, the dynamic binding mode based on switching of the alternative bivalent contacts was previously proposed by us for interaction of LRP1 and FVIII (28). It cannot be excluded that this mechanism may be relevant to interactions of the receptors with majority of their ligands, consistent with the role of RAP as a model ligand of the LDLR family.

The proposed mechanism allows to suggest the structure of the RAP-LRP1 complex considering the molecules' stoichiometry of ≤2:1 (19) and previous models of the complex (46, 57). An early model described simultaneous binding of all three domains of RAP with three different CR doublets of LRP1 (60), and a later model described simultaneous binding of both D1/D2 and D3 of RAP with two CR doublets of LRP1 (57, 60). However, such complexes would have unrealistically superhigh affinities as discussed above. We propose that during biosynthesis, highly flexible exodomain of LRP1 (19) forms a double-twisted spatial string where its clusters II, III, and IV embrace one or two molecules of RAP. Within this compact tunnel-like structure, RAP contacts the CR domains of the receptor in the dynamic bivalent mode. The proposed complex structure is supported by electron microscopy showing that within the complex, LRP1 acquired a “compact-kinked” conformation (19). The proposed complex structure may also be relevant to interactions of RAP and other LDLR family receptors, such as LRP1B and LRP2, which also have four CR clusters as LRP1. In turn, the receptors that have only one CR cluster (LDLR, vLDLR, *etc*.), may form a simpler complex where 1–2 molecules of RAP are statistically “diffused” along the cluster.

The proposed mechanism of RAP and LDLR family interactions, based on dynamic reestablishing of *the same* molecular contacts, resembles mechanisms based on consecutive formation of *new* molecular contacts in such well-known processes as DNA replication, RNA transcription, and protein translation. We believe that such a mechanism is first described here for protein–protein interactions. This mechanism can be universal for majority of interactions of LDLR family receptors with their ligands, in particular, those involved in pathogenesis of hemostasis, type 2 diabetes, obesity, Parkinson's disease, and Alzheimer's disease, while its understanding can facilitate development of medical treatments for those. For example, results of this study allow us to better understand a complex interaction of FVIII and LRP1 (*Chun et al*, under preparation), which can facilitate generation of longer-acting therapeutic FVIII for treatment of Hemophilia A. Future studies will investigate this mechanism in relation to other ligands of the LDLR family receptors.

## Experimental procedures

### Plasmid design

Human LRP1 (Q07954) clusters II, III, and IV, human LDLR (P01130), and human vLDLR (P98155) CR/cluster positions are specified in Table S1. Full-length human RAP (P30533) was synthesized with or without (w/o) insect ER retention signal (sequence: HTEL). All coding sequences were optimized for *Spodoptera frugiperda* using the GeneOptimizer algorithm (61) and synthesized by GenScript.

CR cluster cassettes contain N-terminal HBM (honeybee melittin signal peptide). The cassettes encoding RAP contain N-terminal gp67 secretion signal followed by optimized RAP coding sequence. The tags included for each cassette are described in Table S1.

CR doublets overlapping LRP1 clusters II and IV, and LDLR CR.4–5, including mutated variants of selected doublets, were generated as described (28, 32); *Chun et al.* (manuscript in preparation). The mutagenesis of the conserved tryptophans (W→S) within respective CR doublets is described in Table S1.

Optimized cassettes coding LRP1, LDLR, or vLDLR fragments were cloned into pFastBac-Dual vector (Gibco) under control of the polyhedrin promoter utilizing *SacI/NotI* restrictions sites. Optimized RAP coding sequences were subcloned into pFastBac-Dual carrying clusters utilizing *XhoI/KpnI*. Control plasmids did not contain RAP gene. Generated plasmids were verified by sequencing (GenScript).

### Protein expression and purification

Recombinant baculovirus stocks were generated using Bac-to-Bac system (Invitrogen), following manufacturer recommendations. Optimizations of the expression levels were performed as described previously (62). Sf9 cells (Gibco) were harvested 72 h posttransduction, 200 ml of the culture supernatants was loaded on HisTrap Excel 1 ml (GE Healthcare), followed by column wash with ten column volumes of a high salt/imidazole-EDTA buffer, 20 mM Bis-Tris, 1 M NaCl, 20 mM EDTA, 50 mM imidazole, 0.01% Tween-20, 0.04% NaN_3_, pH 7.4, which resulted in removal of RAP bound to the CR fragments and majority of their multimeric forms. Alternatively, the CR fragments expressed without RAP were washed with a standard medium salt/imidazole buffer, 20 mM Bis-Tris, 500 mM NaCl, 10 mM imidazole, 5 mM CaCl_2_, 0.04% NaN_3_, 0.01% Polysorbate-80, pH 7.4, which resulted in yielding higher abundance of protein multimeric forms at next step. After either type of column wash, the column-bound protein was eluted with 20 mM Bis-Tris, 500 mM NaCl, 500 mM imidazole, 5 mM CaCl_2_, 0.01% Tween-20, 0.04% NaN3, pH 7.4. The eluates were concentrated with Amicon Ultra-4 centrifugal units (10K) and used for size-exclusion chromatography on Superdex 200 Increase 10/300 Gl (GE Healthcare) (for LRP1 clusters, vLDLR, and LDLR) or Superdex 75 Increase 10/300 Gl (GE Healthcare) (for recombinant CRs) column in HBS-P Ca^2+^ pH 7.4 buffer (10 mM HEPES, 150 mM NaCl, 5 mM CaCl_2_, 0.005% Tween-20). CR fragments used for mapping of LRP1 clusters II and IV for binding RAP and for testing interactions with RAP and its fragments in the mutagenic study were generated as described (28). Protein concentrations were determined based on the absorbance at 280 nm using 10 mm cuvette in NanoDrop 2000, the extinction coefficients were calculated based on protein amino acid composition.

### PAGE and western blotting

Proteins were separated in PAGE using Invitrogen Bolt 4–12% Bis-Tris gels (Invitrogen). Western blotting was performed following a standard protocol provided by Li-Cor Biosciences. Briefly, proteins were transferred to a PVDF membrane using iBlot 2 Dry Blotting System (Invitrogen). The membrane was blocked with Protein-Free (PBS) Blocking Buffer (Pierce). The membrane was incubated overnight at 4 °C with primary antibodies diluted 1:1000 in PBST. We used following primary antibodies: rabbit monoclonal to LRPAP1 (Abcam ab76500) and mouse monoclonal antibodies to FLAG (Sigma F3165). Secondary antibodies in dilution 1: 10,000 (anti-Rabbit IRdye@680CW (Li-Cor 925-68071) and anti-mouse IRdye@800CW (Li-Cor 926-32210)) in PBST were incubated for 2 h at room temperature. The fluorescence was detected using Odyssey CLx Imaging System (Li-Cor), data was processed by Image Studio Lite Software (Version 5.2).

### Surface plasmon resonance (SPR)

SPR was performed using Biacore T200 instrument (GE Healthcare). Proteins (recombinant fragments of LDLR family receptors) at concentration of ∼5 ug/ml were covalently coupled to the Series S Sensor Chip CM5 *via* primary amino groups, using the amine-coupling kit (GE Healthcare) with aim level of 250 RU. The blank flow cell was activated and blocked in the absence of protein. As additional controls we used commercially obtained LRP1 fragments: cluster II (recombinant human LRP-1 cluster II Fc chimera protein, R&D, 2368-L2), cluster III (recombinant human LRP-1 cluster III Fc chimera protein, R&D, 4824-L3), cluster IV (recombinant human LRP-1 cluster IV Fc chimera protein, R&D, 5395-L4). The samples, such as recombinant Factor VIII (Advate), RAP (R&D, 4296-LR), recombinant human apolipoprotein E3 Protein (R&D, 4144-AE), were injected at a flow rate 30 ul/min with contact time 180 s, and dissociation 180 s in HBS-P Ca^2+^ pH 7.4 buffer. The samples were injected at different concentrations (n = 5). Chip regeneration was performed by 0.1 M H_3_PO_4_ at a flow rate 50 ul/min with two pulses: 15 s and 30 s. Each test was performed at least twice with proteins that were expressed and purified in biological replicates.

The Biacore T200 evaluation software Version 3.2 (GE Healthcare) was used for analysis of the association and dissociation profiles signals. The K_D_ values were estimated by fitting by nonlinear regression plot of response at equilibrium against the concentration (63).

The experiments for the mapping of RAP-binding sites in LRP1 clusters II and IV and for the mutagenesis study were conducted using Biacore 3000 instrument as described (28). RAP mutant variants were prepared as described (23). For the LRP1 mapping study, we used two sets of the CR doublets from clusters II and IV, expressed and purified independently as described (28).

### Circular dichroism (CD)

Far-UV CD spectra were measured on a Jasco J-815 Spectropolarimeter equipped with a PTC 423S/15 Peltier temperature controller (JASCO Co.) at 25 ± 0.2 °C. The spectral measurements were carried out from 260 nm to 180 nm using a 0.5-mm path length quartz cuvette at a scan speed of 100 nm/min, bandwidth of 1.0 nm, resolution of 0.2 nm, and accumulation of 5. The protein concentration of the samples was adjusted to 10 μM in HBS-Ca^2+^ buffer (10 mM HEPES, 150 mM NaCl, 5 mM CaCl_2_, pH 7.4) or in calcium-deficient Ammonium Sulfate/Potassium Phosphate (ASPP) buffer (10 mM K_2_HPO_4_, 100 mM (NH_4_)_2_SO_4_, pH 6.8). Titration of the samples by EDTA was conducted as described earlier (32). An ellipticity of CD spectra was expressed in millidegrees. For the secondary structure evaluation, CD spectra were analyzed by using CDPro/CONTIN software package (64).

### Modeling of the interactions between receptor-associated protein (RAP) and CR domains

The crystal structure of RAP is available from the Protein Data Bank (PDB 2P01). The CR domains 6–8 and CR domains 7–8 were built using the I-TASSER server (65, 66, 67) (C-score −0.25 and −0.73, respectively), and the top scoring model was used in PyMOL (The PyMOL Molecular Graphics system, Version 4.5 Schrödinger, LLC.) along with PDB 2P01 to create a protein–protein complex as an input structure to use for RosettaPrepack and subsequently RosettaDock (Rosetta 3.11). RosettaDock (51, 52, 53, 54, 55) was used to determine the structure of protein–protein complexes by using rigid body perturbations of the RAP and CR domains (10,000 decoys generated per run). The top-scoring interface-energy models from the initial RosettaDock were then refined further through another run (-dock_pert 1 5). Scores for all models were obtained and the ten best-scoring structures from each of the runs were manually inspected and each unique binding mode is shown in Figure S6, five structures for CR.6–7 and two structures for CR.7–8 were identified. I-TASSER input sequences/results and RosettaDock models are provided as Supplementary material (zip folder).

### Data analysis and statistical rationale

All data points were included in the results. The expression and purification experiments were performed as biological duplicates. The SPR experiments were performed at least biological duplicates. CD experiments were performed with technical duplicates. The data is expressed as average ± SD. The statistical difference was determined by ANOVA tests, the value *p* ≤ 0.05 was set as a significance border. We used Graphpad Prism 9 or Microsoft Excel (Microsoft Office 365 Pro Plus) for statistical calculations.

## Data availability

The data are available within the article. Additional data available upon request, please email Dr. A. Sarafanov (andrey.sarafanov@fda.hhs.gov).

## Supporting information

This article contains supporting information.

## Conflict of interests

The authors declare that they have no conflicts of interest with the contents of this article.
