# Supplementary material for: Molecular chaperone RAP interacts with LRP1 in a dynamic bivalent mode and enhances folding of ligand-binding regions of other LDLR family receptors
Source: J Biol Chem. 2021 May 29;297(1):100842. doi: 10.1016/j.jbc.2021.100842 (PMC8239462; doi:10.1016/j.jbc.2021.100842)
Supplement: Figures S1–S8 and Table S2 [file mmc2.pdf]

Molecular chaperone RAP interacts with LRP1 in a dynamic bivalent mode and enhances folding of ligand-binding regions of other LDLR family receptors

Ekaterina Marakasova, Philip Olivares, Elena Karnaukhova, Haarin Chun, Nancy E. Hernandez, James H. Kurasawa, Gabriela U. Hassink, Svetlana A. Shestopal, Dudley K. Strickland and Andrey G. Sarafanov

Supporting Information

Page

Figure S1. LRP1 complement-type repeats amino acid sequences alignment……......…..…....…..……S-2

Figure S2. Far-UV CD data for expressed LRP1 cluster II, CR.6-8, and CR.30-31 ……………………S-5

Figure S3. Binding of RAP to expressed CR-clusters of LRP1, LDLR, and vLDLR by SPR.……........S-7

Figure S4. Binding of ApoE3 to expressed CR-clusters of LDLR and vLDLR by SPR………....……...S-8

Figure S5. Binding of RAP to expressed LRP1 CR-fragments by SPR……….……….....……......…..S-10

Figure S6. Structure view of the RosettaDock top-scoring interface energy models of interaction of CR-fragments and RAP……………….………………...……………….…….…………………….....…..S-11

Figure S7. RosettaDock decoy interface energy models of interaction of CR-fragments and RAP………......……..……………..……………………………………………………………………S-12

Figure S8. RosettaDock refinement models of interaction of CR-fragments and RAP…….……...…..S-13

Table S2. Purification yields of expressed fragments of LDLR, vLDLR, and LRP1.…………….……...S-14

Cluster I					**1 2 3 4**	**5 67**		*Spacer*
**CR.01**	(0025-0066)	KT**C**SPKQ FA	**C**RDQIT	**C**ISKG	WR**C**DGERD	**C**PDGSDEAPEI	**C**PQ	SKA
**CR.02**	(0070-0110)	QR**C**QPNE HN	**C**LGTEL	**C**VPMS	RL**C**NGVQD	**C**MDGSDEGPH	**C**RE	
Cluster II								
**CR.03**	(0852-0892)	PQ**C**QPGE FA	**C**ANSR	**C**IQER	WK**C**DGDND	**C**LDNSDEAPAL	**C**HQ	
**CR.04**	(0893–0933)	HT**C**PSDR FK	**C**ENNR	**C**IPNR	WL**C**DGDND	**C**GNSEDESNAT	**C**SA	
**CR.05**	(0934–0973)	RT**C**PPNQ FS	**C**ASGR	**C**IPIS	WT**C**DLDDD	**C**GDRSDESAS	**C**AY	
**CR.06**	(0974–1013)	PT**C**FPLTQFT	**C**NNGR	**C**ININ	WR**C**DNDND	**C**GDNSDEAG	**C**S*H*	
**CR.07**	(1013–1053)	*H*S**C**SSTQ FK	**C**NSGR	**C**IPEH	WT**C**DGDND	**C**GDYSDETHAN	**C**TN	*QATRPP*
**CR.08**	(1060–1099)	GG**C**HTDE FQ	**C**RLDGL	**C**IPLR	WR**C**DGDTD	**C**MDSSDEKS	**C**EG	*VT*
**CR.09**	(1060–1099)	HV**C**DPSVKFG	**C**KDSAR	**C**ISKA	WV**C**DGDND	**C**EDNSDEEN	**C**ES	
**CR.10**	(1143–1184)	LA**C**RPPSHP	**C**ANNTSV	**C**LPPD	KL**C**DGNDD	**C**GDGSDEGEL	**C**DQ	
Cluster III								
**CR.11**	(2522–2563)	SS**C**RAQDEFE	**C**ANGE	**C**INFSLT	**C**DGVPH	**C**KDKSDEKPSY	**C**NS	
**CR.12**	(2564–2602)	RR**C**KKT FRQ	**C**SNGR	**C**VSNMLW	**C**NGADD	**C**GDGSDEIP	**C**NK	
**CR.13**	(2603–2641)	TA**C**GVGE FR	**C**RDGT	**C**IGNS	SR**C**NQFVD	**C**EDASDEMN	**C**SA	
**CR.14**	(2642–2690)	TD**C**SSYFRLGVKGVLFQP	**C**ERTSL	**C**YAPS	WV**C**DGAND	**C**GDYSDERD	**C**PG	*VKR*
**CR.15**	(2694–2732)	PR**C**PLNY FA	**C**PSGR	**C**IPMS	WT**C**DKEDD	**C**EHGEDETH	**C**N*K*	
**CR.16**	(2732–2771)	*K*F**C**SEAQ FE	**C**QNHR	**C**ISKQ	WL**C**DGSDD	**C**GDGSDEAAH	**C**EG	
**CR.17**	(2772–2814)	KT**C**GPSS FS	**C**PGTHV	**C**VPER	WL**C**DGDKD	**C**ADGADESIAAG	**C**LY	*N*
**CR.18**	(2816–2855)	ST**C**DDRE FM	**C**QNRQ	**C**IPKH	FV**C**DHDRD	**C**ADGSDESPE	**C**EY	
**CR.19**	(2856–2899)	PT**C**GPSE FR	**C**ANGR	**C**LSSRQ	WE**C**DGEND	**C**HDQSDEAPKNPH	**C**TS	*QE*
**CR.20**	(2902–2951)	HK**C**NASSQFL	**C**SSGR	**C**VAEA	LL**C**NGQDD	**C**GDSSDERG	**C**HI	
Cluster IV								
**CR.21**	(3332–3371)	SN**C**TASQ FV	**C**KNDK	**C**IPFW	WK**C**DTEDD	**C**GDHSDEPPD	**C**PE	
**CR.22**	(3372–3410)	FK**C**RPGQ FQ	**C**STGI	**C**TNPA	FI**C**DGDND	**C**QDNSDEAN	**C**DI	
**CR.23**	(3411–3450)	HV**C**LPSQ FK	**C**TNTNR	**C**IPGI	FR**C**NGQ DN	**C**GDGEDERD	**C**PE	
**CR.24**	(3451–3491)	VT**C**APNQ FQ	**C**SITKR	**C**IPRV	WV**C**DRDND	**C**VDGSDEPAN	**C**TQ	
**CR.25**	(3492–3533)	MT**C**GVDE FR	**C**KDSGR	**C**IPAR	WK**C**DGEDD	**C**GDGSDEPKEE	**C**DE	
**CR.26**	(3534–3572)	RT**C**EPYQ FR	**C**KNNR	**C**VPGR	WQ**C**DYDND	**C**GDNSDEES	**C**TP	
**CR.27**	(3573–3611)	RP**C**SESE FS	**C**ANGR	**C**IAGR	WK**C**DGDHD	**C**ADGSDEKD	**C**T*P*	
**CR.28**	(3612–3649)	*P*R**C**DMDQ FQ	**C**KSGH	**C**IPLR	WR**C**DADAD	**C**MDGSDEEA	**C**GT	*GV*
**CR.29**	(3652–3692)	RT**C**PLDE FQ	**C**NNTL	**C**KPLA	WK**C**DGEDD	**C**GDNSDENPEE	**C**AR	
**CR.30**	(3693–3733)	FV**C**PPNRPFR	**C**KNDRV	**C**LWIG	RQ**C**DGTDN	**C**GDGTDEED	**C**EP	*PTAHT*
**CR.31**	(3739–3778)	TH**C**KDKKEFL	**C**RNQR	**C**LSSS	LR**C**NMFDD	**C**GDGSDEED	**C**SI	

Figure S1. LRP1 complement-type repeats amino acid sequences alignment. The LRP1 sequence (**Q07954**) is aligned by six conserved cysteines within each CR. Other conserved residues (shadowed) at positions 1 and 3, coordinate Ca^{2+} via backbone carbonyl oxygen, and at positions 2, 4, 6, and 7, coordinate Ca^{2+} via side-chain carboxyl oxygen (*Fass et al*, 1997); the conserved D at position 5 can coordinate Ca^{2+} alternatively to D at position 4 (*Guo et al*, 2004). During ligand binding, the conserved W, or less conserved F, at position 1, interacts with the aliphatic portion of a “critical” lysine of the ligand, and the conserved acidic residues interact with the lysine’s positively charged portion (*Fisher et al*, 2006). Residues shown in *italic* within CRs 6-7 (H-1013), CRs 15-16 (K-2732), and CRs 27-28 (P-3611) are shared between the adjacent CRs, thus shown twice in the adjacent lanes.

A **HBS-Ca^{2+} buffer**

Cluster II

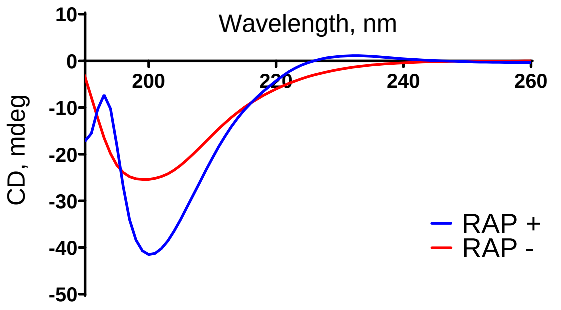

B **HBS-Ca^{2+} buffer**

Cluster II (RAP+)

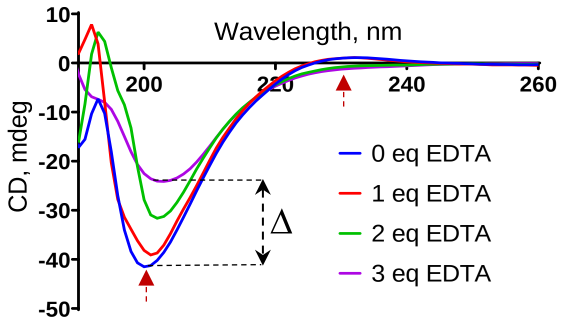

C **HBS-Ca^{2+} buffer**

Cluster II (RAP-)

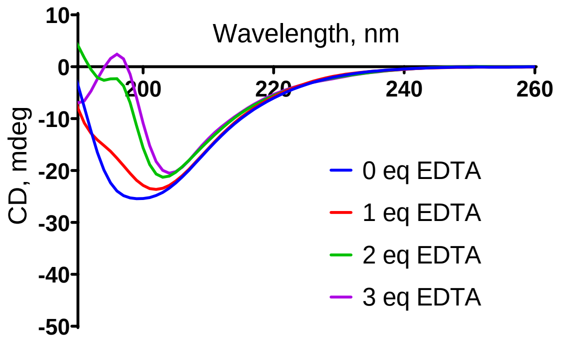

H **ASPP buffer**

Cluster II

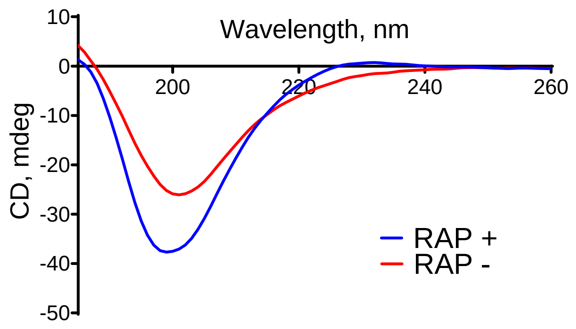

I **ASPP buffer**

Cluster II (RAP+)

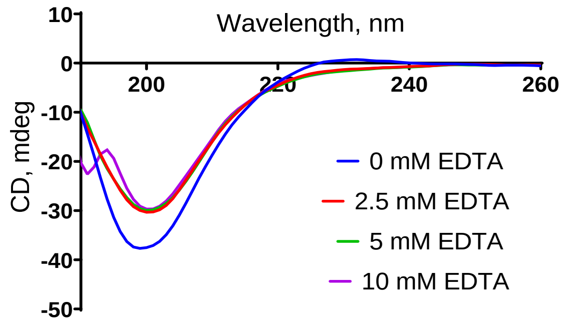

J **ASPP buffer**

Cluster II (RAP-)

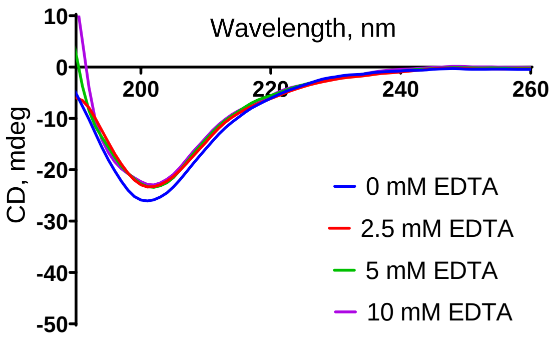

D **HBS-Ca^{2+} buffer**

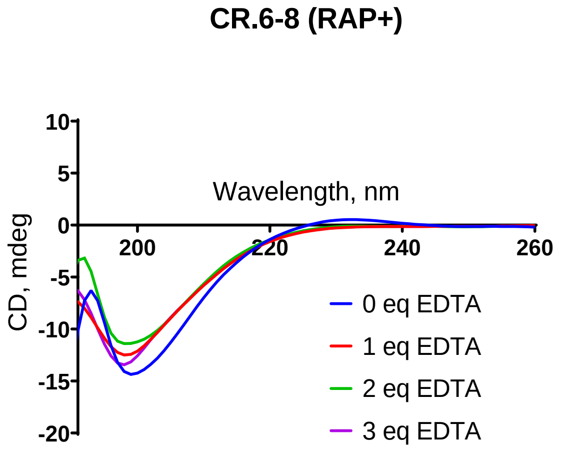

K **ASPP buffer**

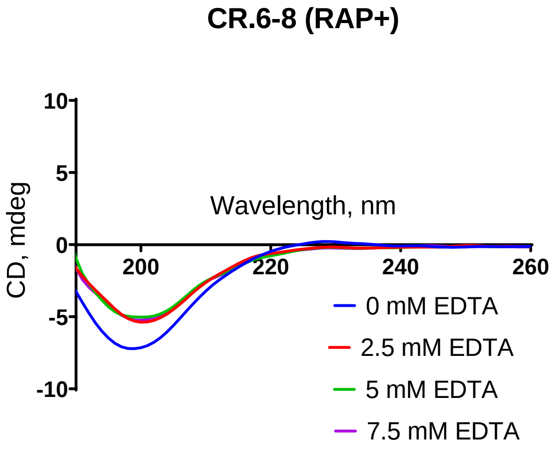

E **HBS-Ca^{2+} buffer**

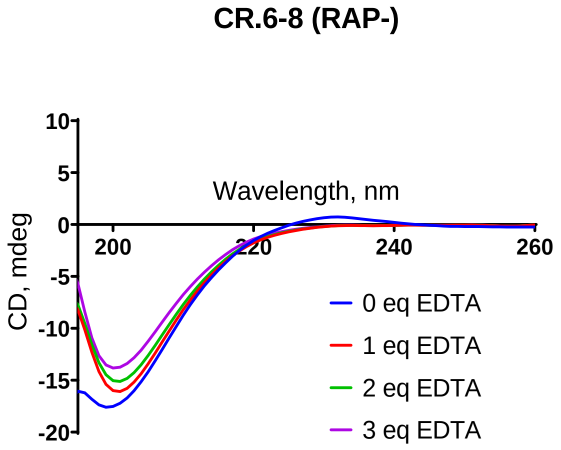

L **ASPP buffer**

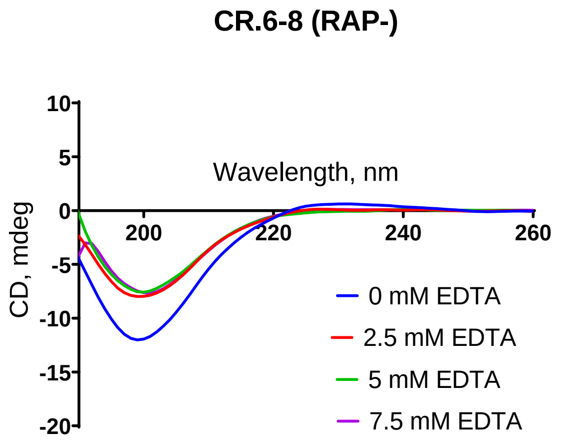

F **HBS-Ca^{2+} buffer**

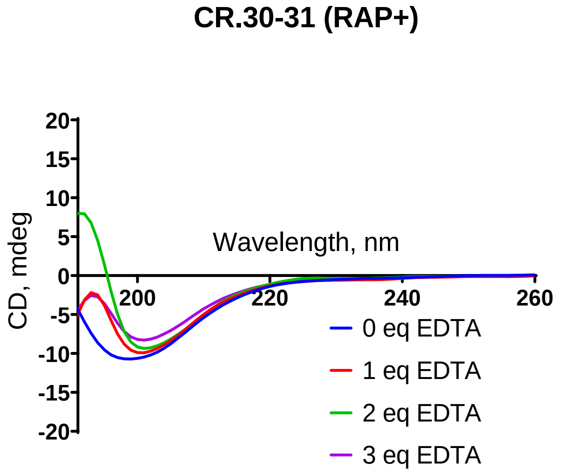

M **ASPP buffer**

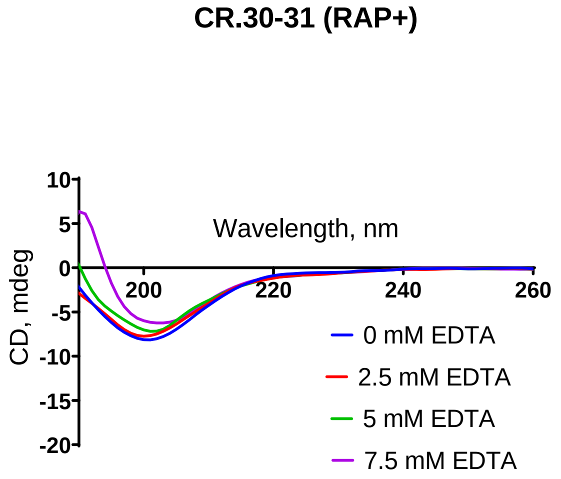

G **HBS-Ca^{2+} buffer**

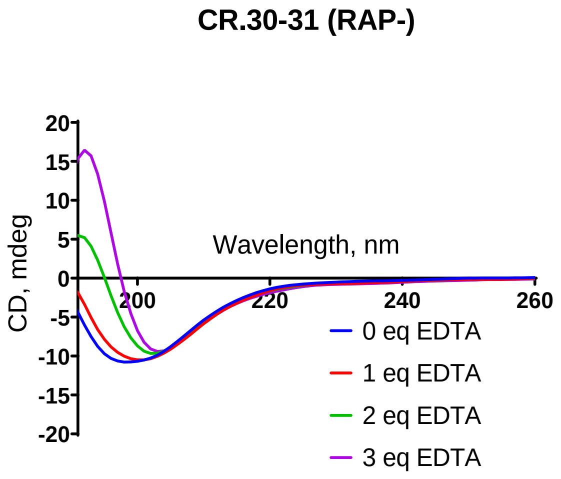

N **ASPP buffer**

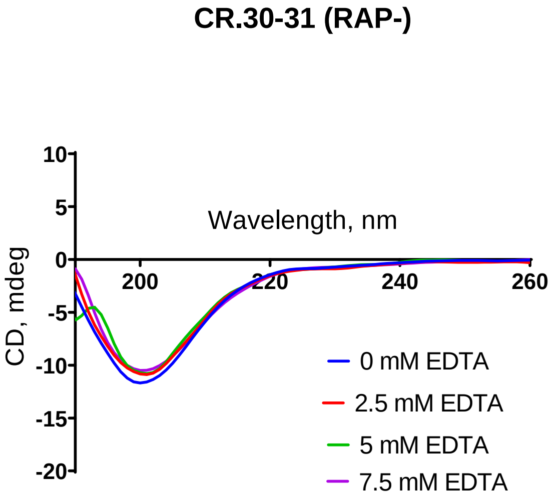

Figure S2. Far-UV CD data for expressed LRP1 cluster II, CR.6-8, and CR.30-31. The left column (*A*–*G*) shows testing of proteins in HBS-Ca^{2+} buffer containing 5 mM Ca^{2+}, and the right column (*H*–*N*) shows results collected in Ammonium Sulfate/Potassium Phosphate (ASPP) buffer that does not contain Ca^{2+}; in all samples, the proteins were at concentrations of 10 mM. Concentration of added EDTA is shown in molar equivalent of calcium in samples in HBS-Ca^{2+} buffer, or in molar concentration (mM) for samples in ASPP buffer.

A, *B*, *H*, and *I*, the spectra of cluster II expressed with (RAP+) and *C* and *J*, the spectra of the cluster expressed without RAP (RAP-). *B and I*, EDTA titration of cluster II expressed with (RAP+); *C* and *J*, titration of protein expressed without RAP (RAP-).

D and *K*, EDTA titration of CR.6-8 expressed with (RAP+); *E* and *L*, titration of protein expressed without RAP (RAP-).

F and *M*, EDTA titration of CR.30-31 (negative control) expressed with (RAP+); *G* and *N*, titration of protein expressed without RAP (RAP-).

In panel *B*, red arrows show negative peak at ~200 nm and a band at ~230 nm. Δ is a change in intensity of the negative peak at ~200 nm upon the addition of EDTA to maximal concentration (3 eq EDTA).

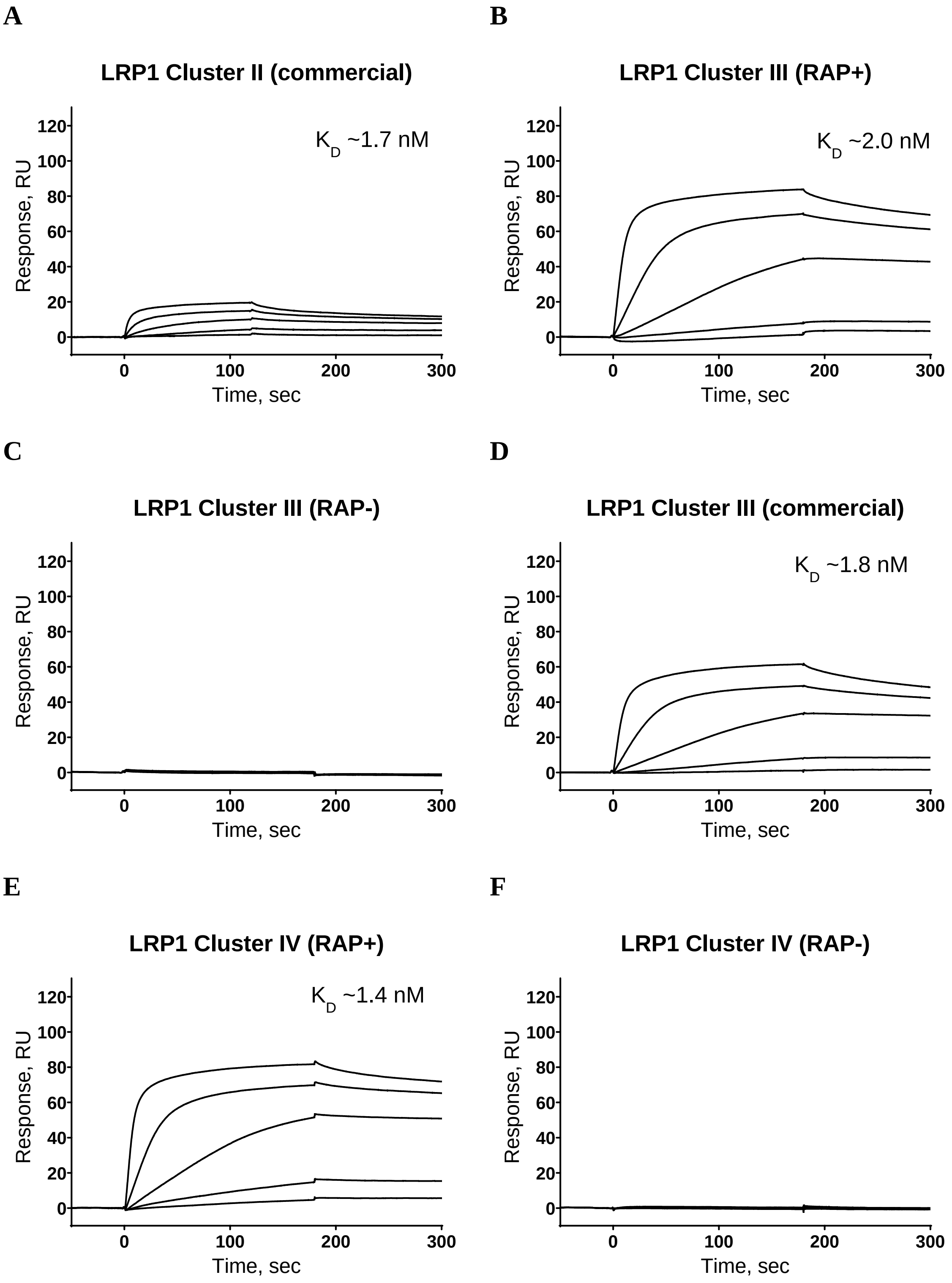
A
LRP1 Cluster II (commercial)
K_D ~1.7 nM
Response, RU
Time, sec
B
LRP1 Cluster III (RAP+)
K_D ~2.0 nM
Response, RU
Time, sec
C
LRP1 Cluster III (RAP-)
Response, RU
Time, sec
D
LRP1 Cluster III (commercial)
K_D ~1.8 nM
Response, RU
Time, sec
E
LRP1 Cluster IV (RAP+)
K_D ~1.4 nM
Response, RU
Time, sec
F
LRP1 Cluster IV (RAP-)
Response, RU
Time, sec

G

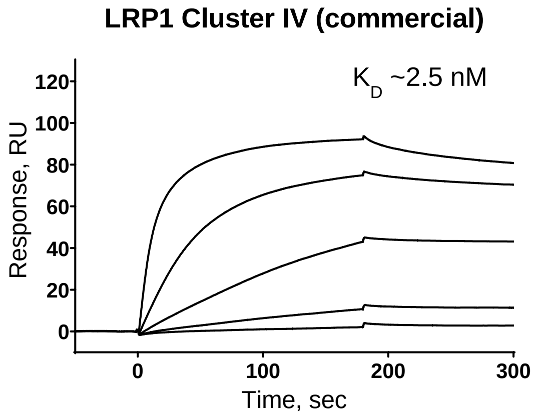

H

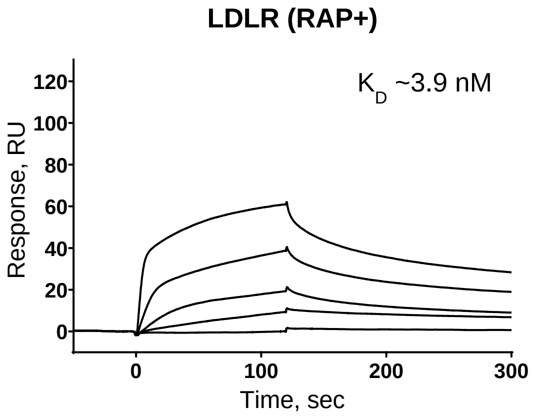

I

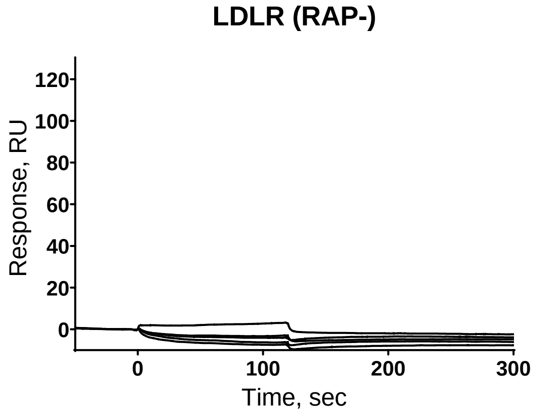

J

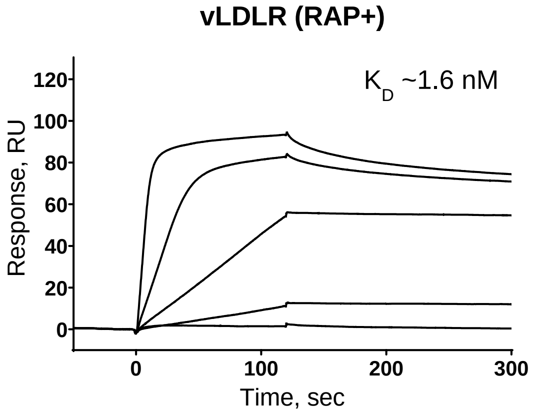

K

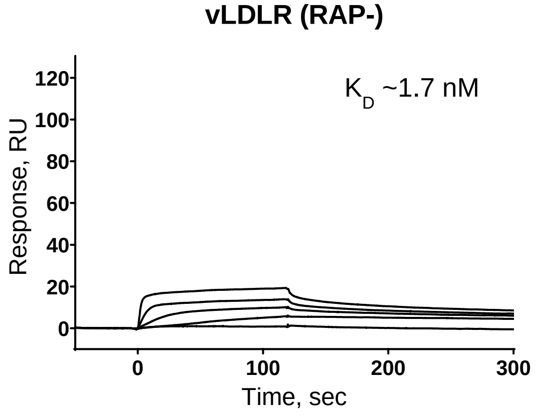

Figure S3. Binding of RAP to expressed CR-clusters of LRP1, LDLR, and vLDLR by SPR. The CR-clusters of: LRP1 (*B*, *C*, *E*, and *F*), LDLR (*H* and *I*), or vLDLR *(J* and *K*) , expressed with RAP (RAP+) or without RAP (RAP-), or commercial LRP1 clusters II-IV (control) (*A*, *D*, and *G*) were immobilized on a S series CM5 chip by amine-coupling with aim level of 250 RU and tested for binding with RAP (commercial) injected at 0.08 nM, 0.31 nM, 1.25 nM, 5 nM, or 20 nM. The K_Ds were calculated using a steady-state affinity model as average from two independent experiments (*Methods*). The K_D values shown correspond to fitting the binding curves in respective representative experiments.

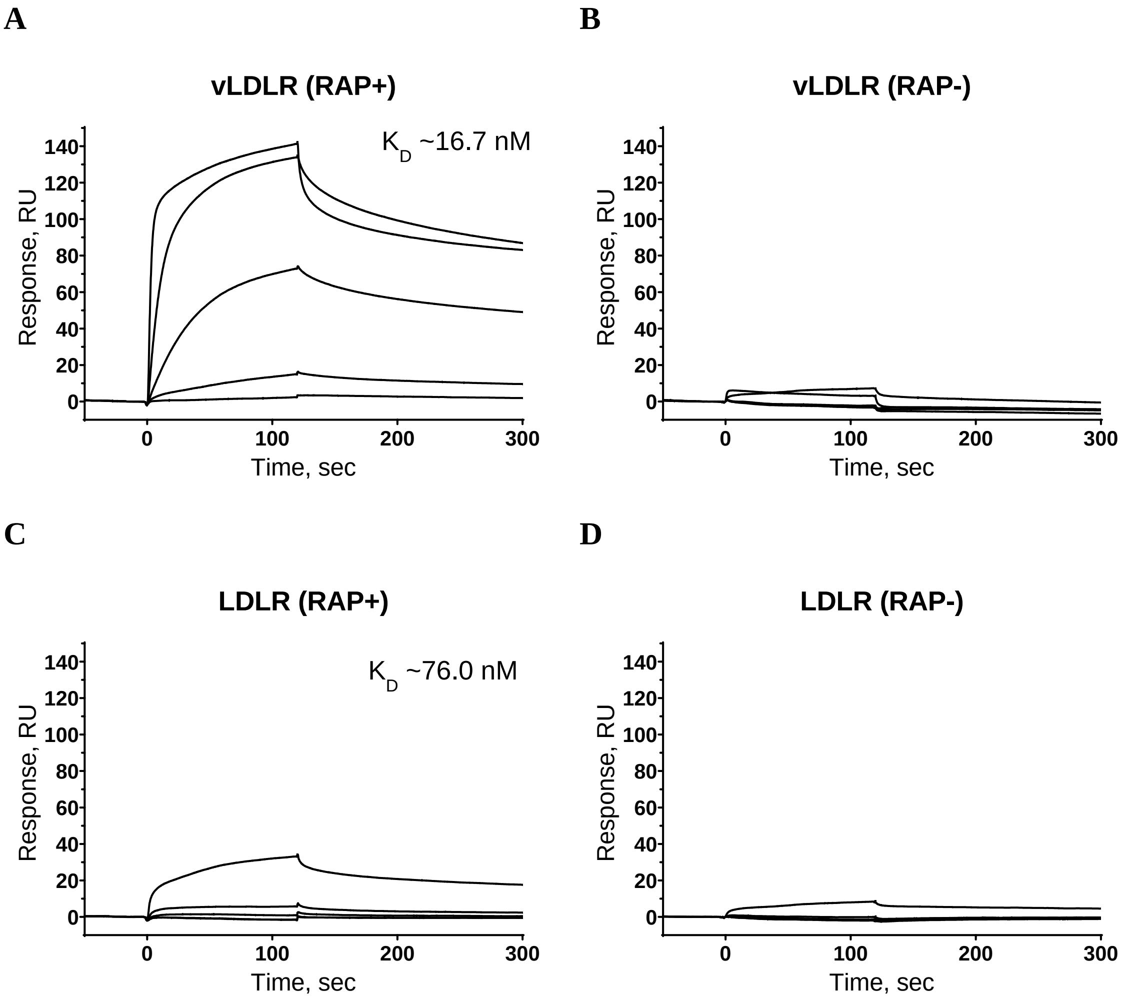

Figure S4. Binding of ApoE3 to expressed CR-clusters of LDLR and vLDLR by SPR. The CR-clusters of LDLR and vLDLR expressed with RAP (RAP+) or without RAP (RAP-) were immobilized on a S series CM5 chip by amine-coupling with aim level of 250 RU and tested for binding with ApoE3 injected at 0.78 nM, 3.13 nM, 12.5 nM, 50 nM, or 200 nM. The K_Ds were calculated using a steady-state affinity model as average from two independent experiments (*Methods*). The K_D values shown correspond to fitting the binding curves in respective representative experiments.

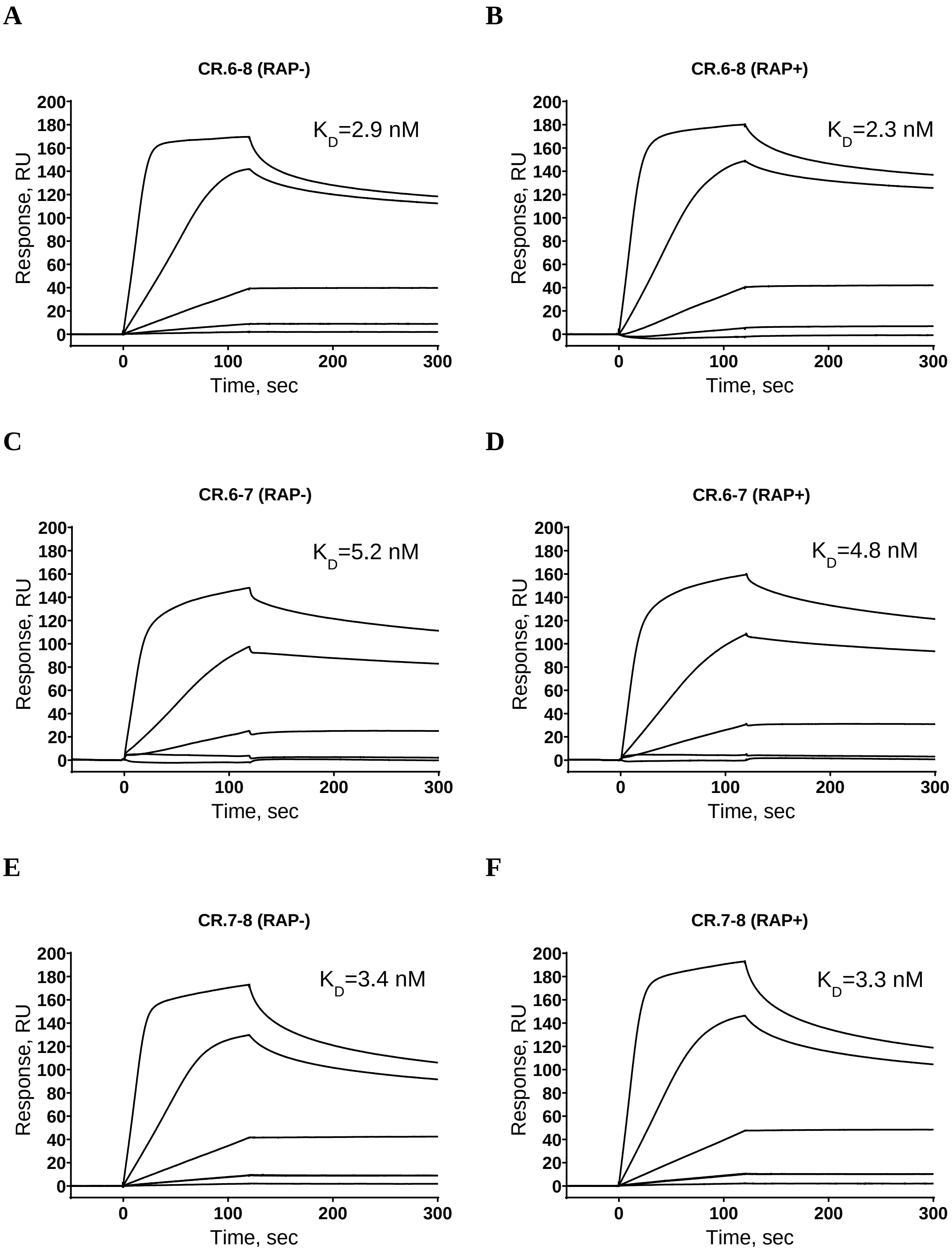
A
CR.6-8 (RAP-)
K_D=2.9 nM
Response, RU
Time, sec
B
CR.6-8 (RAP+)
K_D=2.3 nM
Response, RU
Time, sec
C
CR.6-7 (RAP-)
K_D=5.2 nM
Response, RU
Time, sec
D
CR.6-7 (RAP+)
K_D=4.8 nM
Response, RU
Time, sec
E
CR.7-8 (RAP-)
K_D=3.4 nM
Response, RU
Time, sec
F
CR.7-8 (RAP+)
K_D=3.3 nM
Response, RU
Time, sec

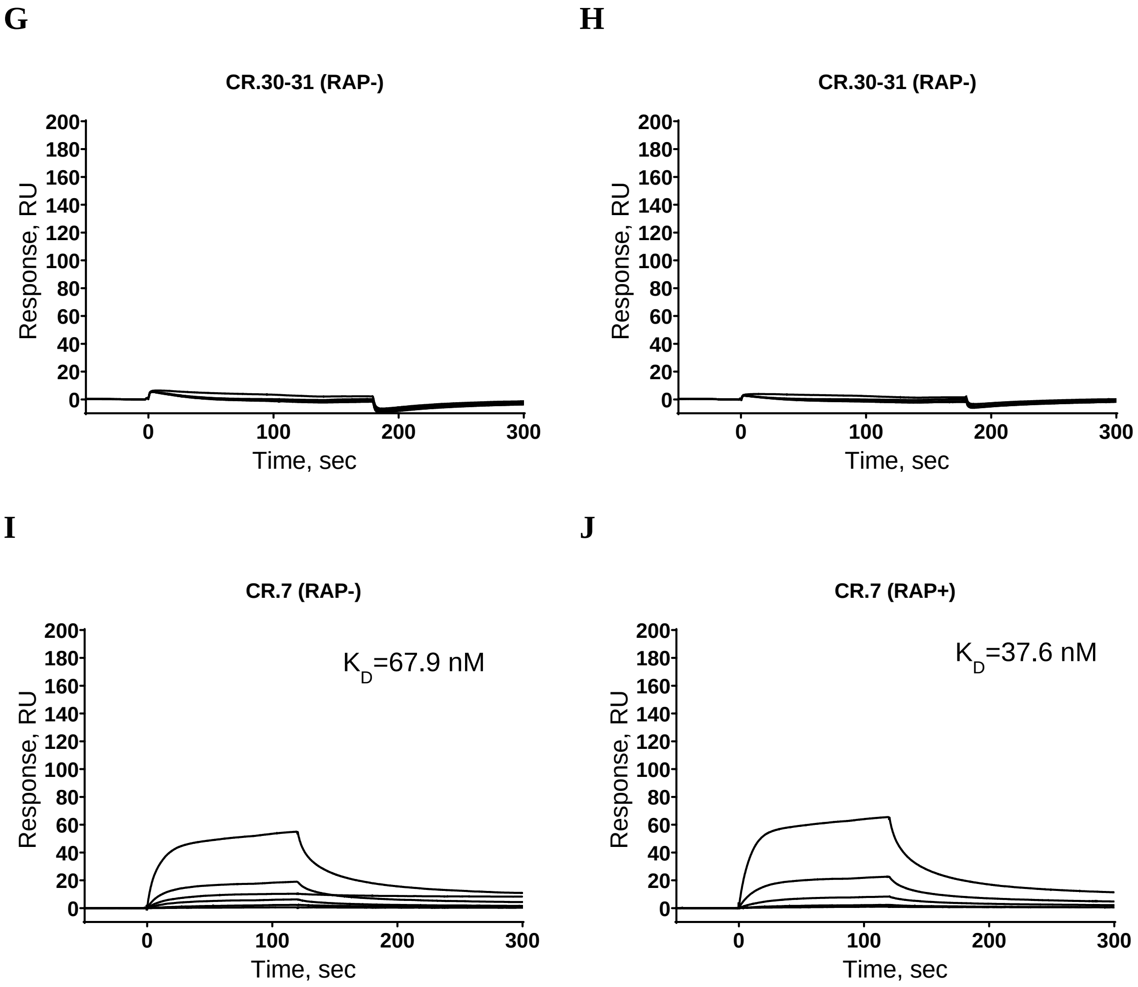

Figure S5. Binding of RAP to expressed LRP1 CR-fragments by SPR. LRP1 fragments: CR.6-8 (*A* and *B*), CR.6-7 (*C* and *D*), CR.7-8 (*E* and *F*), CR.30-31 (*G* and *H*), and CR.7 (*I* and *J*) expressed with RAP (RAP+) or without RAP (RAP-) were immobilized on a S series CM5 chip by amine-coupling with aim level of 250 RU and tested for binding with RAP (commercial) injected at 0.08 nM, 0.31 nM, 1.25 nM, 5 nM, or 20 nM. The K_Ds were calculated using a steady-state affinity model as average from two independent experiments (*Methods*). The K_D values shown correspond to fitting the binding curves in respective representative experiments.

Interactions of RAP and CR.6-8 triplet

A

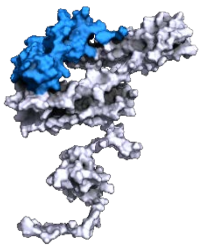

B

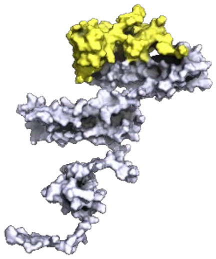

C

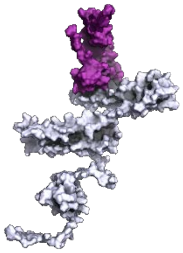

D

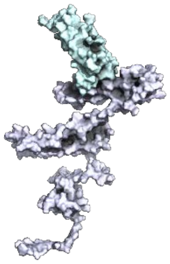

E

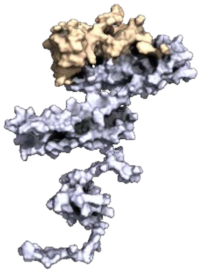

Interactions of RAP and CR.7-8 (doublet)

F

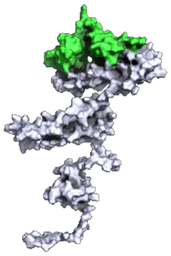

G

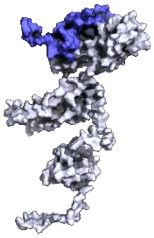

Domain structure of RAP

H

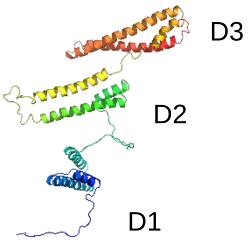

Figure S6. Structure view of the RosettaDock top-scoring interface energy models of interaction of CR-fragments and RAP. *A-E*, top-scoring models demonstrating interaction between CR.6-8 triplet (colored surface) and RAP (grey color). *F-G*, top-scoring models demonstrating interaction between CR.7-8 doublet (colored surface) and RAP (grey color). *H*, RAP structure colored in rainbow, indicated the domains as D1, D2, and D3. The used structure of RAP was taken from the Protein Data Bank (PDB 2P01), and the structures of CR.6-8 and CR.7-8 were built using the I-TASSER server (Methods).

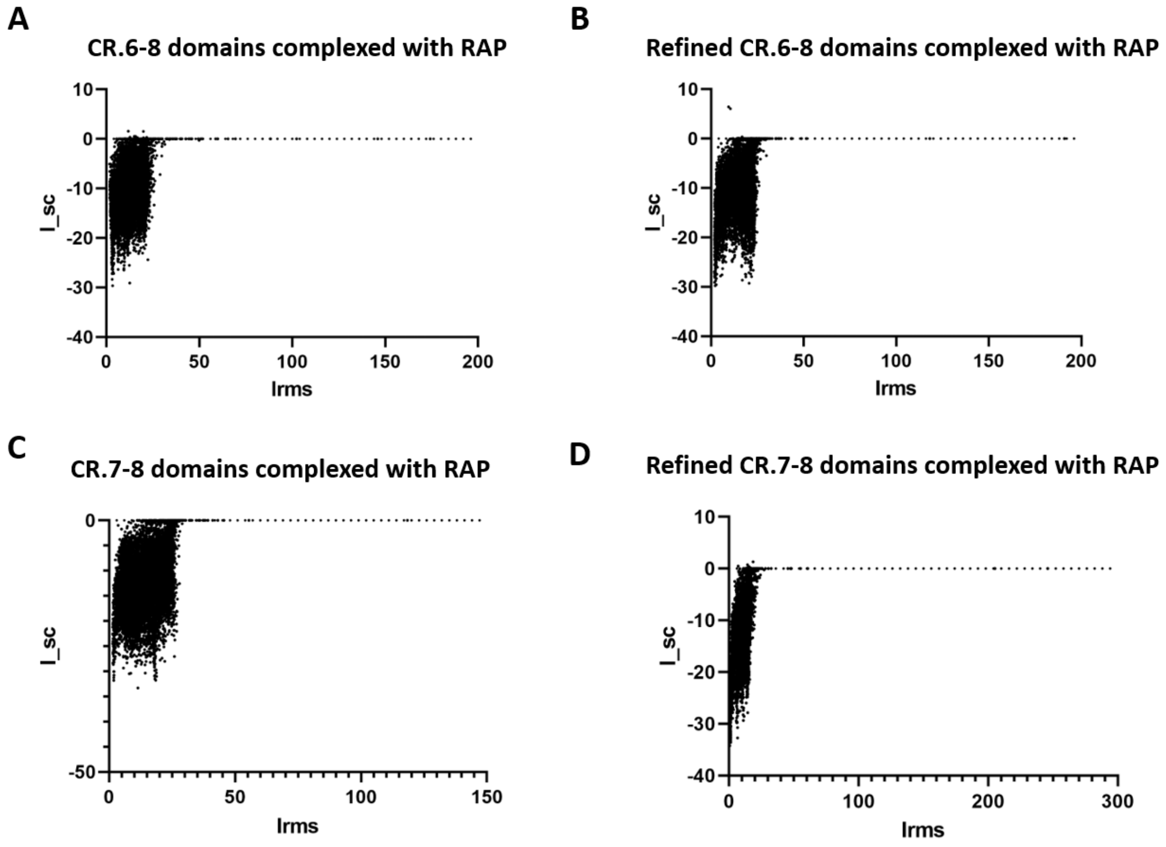

Figure S7. RosettaDock decoy interface energy models of interaction of CR-fragments and RAP. All interface energy scores depict energy funnels (*A*-*B*) RosettaDock decoy interface energy (I_sc) vs interface root-mean-square deviation (Irms) plots for CR.6-8 triplet complexed with RAP. *C*-*D*, interface energy for CR.7-8 doublet complexed with RAP. The used structure of RAP was taken from the Protein Data Bank (PDB 2P01), and the structures of CR.6-8 and CR.7-8 were built using the I-TASSER server (Methods).

A

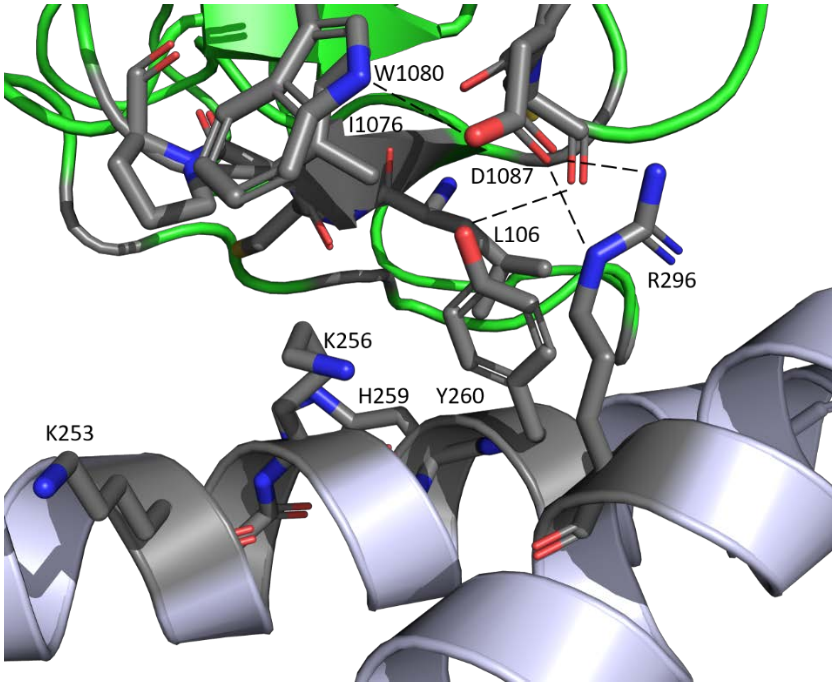

B

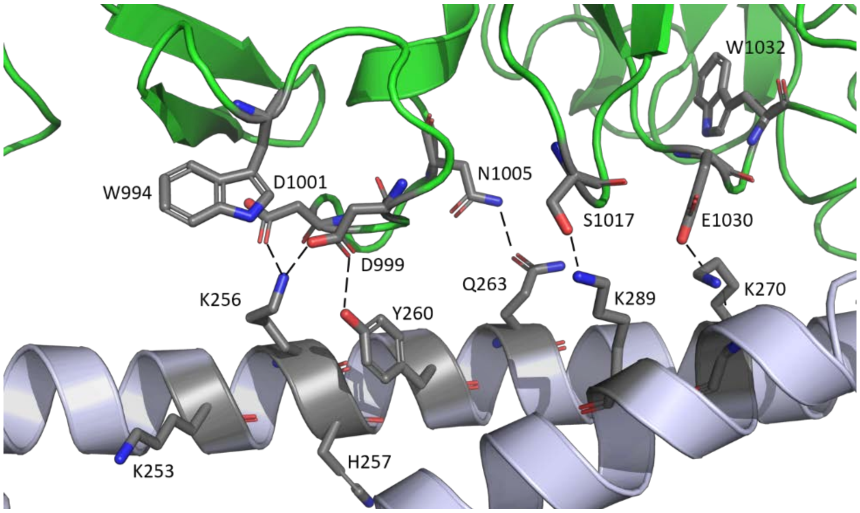

Figure S8. RosettaDock refinement models of interaction of the CR-fragments and RAP. *A*, a single binding mode interaction of residues around CR.8 W1080 having favorable interactions with RAP D3 domain. *B*, a bidentate binding mode interaction with the residues around CR.6 W994 and CR.7 W1032 interacting with RAP D3 domain residues. The used structure of RAP was taken from the Protein Data Bank (PDB 2P01), and the structures of CR.6-8 and CR.7-8 were built using the I-TASSER server (Methods).

Table S2. Purification yields of expressed fragments of LDLR, vLDLR, and LRP1

Protein	RAP co-expression (Yes/No)[2]	Purification yield[1] (μg/L)[3]
LDLR	Yes	1403
LDLR cluster	No	774
vLDLR cluster	Yes	555
vLDLR cluster	No	164
LRP1 cluster II	Yes	810
LRP1 cluster II	No	389
LRP1 cluster III	Yes	2431
LRP1 cluster III	No	621
LRP1 cluster IV	Yes	850
LRP1 cluster IV	No	238
LRP1 CR.6-8	Yes	1804
LRP1 CR.6-8	No	488
LRP1 CR.6-7	Yes	2998
LRP1 CR.6-7	No	1230
LRP1 CR.7-8	Yes	2306
LRP1 CR.7-8	No	3539
LRP1 CR.30-31	Yes	289
LRP1 CR.30-31	No	312
LRP1 CR.7	Yes	2140
LRP1 CR.7	No	3448

[1]Shown for representative experiments

[2]*Yes*, expressed with RAP; *No*, expressed without RAP

[3]Per liter of expression media
